# *Helicobacter pylori*-infected human neutrophils exhibit impaired chemotaxis and a uropod retraction defect

**DOI:** 10.3389/fimmu.2022.1038349

**Published:** 2022-10-20

**Authors:** Allan Prichard, Lisa Khuu, Laura C. Whitmore, Daniel Irimia, Lee-Ann H. Allen

**Affiliations:** ^1^ Department of Microbiology and Immunology, University of Iowa, Iowa City, IA, United States; ^2^ Department of Molecular Microbiology and Immunology, University of Missouri, Columbia, MO, United States; ^3^ Department of Medicine, Division of Infectious Diseases, University of Iowa, Iowa City, IA, United States; ^4^ Department of Surgery, BioMEMS Resource Center, Massachusetts General Hospital, Harvard Medical School, Boston, MA, United States; ^5^ Iowa City VA Healthcare System, Iowa City, IA, United States; ^6^ Harry S. Truman Memorial VA Hospital, Columbia, MO, United States

**Keywords:** neutrophils, chemotaxis, uropod, actin, ROCK, RhoA, myosin II, *H. pylori*

## Abstract

*Helicobacter pylori* is a major human pathogen that colonizes the gastric mucosa and plays a causative role in development of peptic ulcers and gastric cancer. Neutrophils are heavily infected with this organism *in vivo* and play a prominent role in tissue destruction and disease. Recently, we demonstrated that *H. pylori* exploits neutrophil plasticity as part of its virulence strategy eliciting N1-like subtype differentiation that is notable for profound nuclear hypersegmentation. We undertook this study to test the hypothesis that hypersegmentation may enhance neutrophil migratory capacity. However, EZ-TAXIScan™ video imaging revealed a previously unappreciated and progressive chemotaxis defect that was apparent prior to hypersegmentation onset. Cell speed and directionality were significantly impaired to fMLF as well as C5a and IL-8. Infected cells oriented normally in chemotactic gradients, but speed and direction were impaired because of a uropod retraction defect that led to cell elongation, nuclear lobe trapping in the contracted rear and progressive narrowing of the leading edge. In contrast, chemotactic receptor abundance, adhesion, phagocytosis and other aspects of cell function were unchanged. At the molecular level, *H. pylori* phenocopied the effects of Blebbistatin as indicated by aberrant accumulation of F-actin and actin spikes at the uropod together with enhanced ROCKII-mediated phosphorylation of myosin IIA regulatory light chains at S19. At the same time, RhoA and ROCKII disappeared from the cell rear and accumulated at the leading edge whereas myosin IIA was enriched at both cell poles. These data suggest that *H. pylori* inhibits the dynamic changes in myosin IIA contractility and front-to-back polarity that are essential for chemotaxis. Taken together, our data advance understanding of PMN plasticity and *H. pylori* pathogenesis.

## Introduction

Human polymorphonuclear leukocytes (PMNs, neutrophils) are the most abundant circulating white blood cell type and are vital to host defense as they are rapidly recruited to sites of infection where they destroy invading bacteria and fungi through a combination of phagocytosis, oxidant production, granule mobilization and release of extracellular traps (1–4). In addition to cytoplasmic granules, neutrophils are distinguished morphologically by their nuclei which are comprised of three or four interconnected lobes, a structure that has been suggested to aid cell migration through tight spaces and tissue junctions (5). To reach sites of infection PMNs follow chemical gradients of chemoattractants of host and microbial origin such as C5a, formyl peptides and IL-8 (CXCL8) (6). Critical to this response are G protein-coupled receptors at the neutrophil surface. During attractant sensing, receptor activation induces signals that lead to coordinated front-to-back cell polarization and directed migration (7, 8).

Two general modes of cell migration have been described. Mesenchymal migration of fibroblasts is relatively slow and is driven by towing from the front. Conversely, rapid amoeboid migration of neutrophils is driven by contraction of the rear uropod which pushes the cell body forward (9). Critical to this process are distinct zones of ‘frontness’ and ‘backness’ that are established and maintained by differential localization and activation of Rho family GTPases and their downstream targets. Notably, Rac1 regulates actin polymerization and lamellipodium formation at the cell front, whereas RhoA localizes at the cell rear and regulates actomyosin-based contraction that is essential for uropod detachment and retraction. Critical players in this process include Rho kinase II (ROCKII) and non-muscle myosin IIA (9–13). Thus, defects in RhoA, ROCKII or myosin IIA abundance or activity disrupt neutrophil chemotaxis by inhibiting uropod retraction (10, 14–16).

*Helicobacter pylori* is a Gram-negative bacterial pathogen of humans that thrives in the gastric mucosa and plays a causative role in development of peptic ulcers and gastric cancer (17). Once acquired, this bacterium typically persists for the lifetime of the host and this infection is notable for eliciting a chronic, neutrophil-dominant inflammatory response that enhances tissue damage but does not eliminate the pathogen (18, 19). Host and bacteria factors including IL-8, the *H. pylori* neutrophil-activating protein (NapA), urease, and the Hp(2–20) cecropin-like peptide collaborate to recruit neutrophils to the stomach, and electron microscopy analysis of patient biopsies demonstrates that neutrophils traverse the gastric epithelium in large numbers and are heavily infected in this locale (18, 20). Phagocytosis of bacteria occurs by non-opsonic mechanisms and triggers a robust respiratory burst that is coupled to extracellular rather than intracellular release of toxic reactive oxygen species (ROS) (18, 19, 21). Oxidant-mediated tissue damage facilitates bacterial nutrient acquisition and persistence, and it is established that PMN density correlates directly with disease severity (19).

Since the pioneering studies of Fridlender and colleagues, it has become clear that human and murine neutrophils are not homogenous and instead exhibit significant functional and phenotypic heterogeneity in different microenvironments and disease states (22–28). Although studies of neutrophil plasticity are advancing rapidly, our understanding of the underlying mechanisms and functional consequences is incomplete. Recently, we demonstrated that *H. pylori* elicits N1-like subtype differentiation of human neutrophils (29). These cells are proinflammatory and cytotoxic, CD62L^low^, CD11b^bright^, CD66b^bright^, CD63^bright^, and are notable for progressive elaborate nuclear hypersegmentation that is apparent in small numbers of cells at six hours and progresses thereafter such that infected cells exhibit up to 19 nuclear lobes each by 24-36 hours (29). As nuclear segmentation is generally believed to facilitate PMN migration (5), we hypothesized that this would be enhanced by *H. pylori*-induced changes in nuclear morphology.

Herein, we used multiple imaging strategies to interrogate this hypothesis and discovered that prior to hypersegmentation onset chemotaxis of infected neutrophils was significantly impaired whereas other aspects of cell function were unchanged. Impaired migration was attributable to a uropod retraction defect and dysregulated phosphorylation of myosin IIA. These data distinguish *H. pylori*-infected cells from other neutrophil subpopulations described to date and we speculate that impaired migratory capacity may in part account for the fact that this infection remains confined to the gastric mucosa.

## Materials and methods

### Isolation of human neutrophils

Heparinized venous blood of healthy adult volunteers who provided written informed consent was obtained using protocols approved by the Institutional Review Boards of the University of Iowa (200307026 and 201609850) and the University of Missouri (2031144 and 2033122). Neutrophils were purified from heparinized whole blood by dextran sedimentation and Ficoll-Hypaque gradient separation followed by hypotonic lysis of erythrocytes (30). Isolated cells were 90-95% pure with eosinophils as the major contaminant and were suspended at 1 x 10^6^/mL in HEPES-buffered RPMI-1640 medium (Lonza, Walkersville, MD) supplemented with 10% heat-inactivated (HI)-FBS (Gibco, Grand Island, NY) and 2 mM L-glutamine (Lonza). Replicate experiments utilized cells from different donors.

### Cultivation of bacteria and neutrophil infection

*H. pylori* strain NCTC11637 (ATCC #43504) was grown on trypticase soy agar (Difco, Franklin Lakes, NJ) containing 5% defibrinated sheep blood (Hemostat, Dixon, CA) and supplemented with an antibiotic cocktail (5 µg/mL cefsulodin, 5 µg/mL trimethoprim, 10 µg/mL vancomycin) (all from Millipore Sigma, Burlington, MA). Isogenic *H. pylori* mutant strains Δ*cagA*, Δ*cagE*, Δ*napA*, Δ*ureAB*, and Δ*vacA* have been described (29, 31) and were grown on blood agar supplemented with either 25 µg/mL chloramphenicol or 25 µg/mL kanamycin (both Millipore Sigma), as appropriate. After incubation under humidified microaerophilic conditions (5% O_2_, 10% CO_2_ and 85% N_2_) at 37°C for 24 hours, *H. pylori* were collected and washed twice with Hank’s buffered saline solution without calcium and magnesium (HBSS -/-) (Thermo Fisher Scientific, Waltham, MA) containing 1mM D-glucose and were quantified by measurement of the absorbance at 600nm.

*Francisella tularensis* Live Vaccine Strain (LVS) (32–34) was grown on cysteine heart agar (Difco) supplemented with 9% sheep blood (CHAB) and incubated for 48 hours at in a humidified 37°C, 5% CO_2_ incubator. Broth cultures of LVS were started at an OD_600_ of 0.01 in Bacto brain heart infusion (BHI) broth adjusted to 6.8 pH (BD Biosciences, San Jose, CA) and incubated overnight with shaking at 200 rpm. The next morning cultures were diluted to an OD_600_ of 0.2 in BHI broth and incubated with shaking at 200 rpm for 2-4 hours. Mid-exponential phase bacteria were harvested, washed with HBSS containing calcium and magnesium (HBSS+/+) (Thermo Fisher Scientific) and quantified by measurement of the absorbance at 600 nm.

For infection, PMNs were suspended at a concentration of 1 x 10^6^/mL and *H. pylori* were added at a 5:1 multiplicity of infection (MOI) (29, 35). Cultures of 1-2 mL each in snap-cap polypropylene tubes were incubated at 37°C under humidified microaerophilic conditions for the amounts of time indicated below. For infection with *F. tularensis* LVS, PMNs were diluted in HEPES-buffered RPMI-1640 without serum at 5 x 10^6^/mL and infected at an MOI of 200:1 (34). Cultures were incubated at in a humidified 37°C incubator in an atmosphere of 5% CO_2_ in air.

### Assays of neutrophil migration

#### EZ-TAXIScan™ live cell imaging

Neutrophils were suspended at 1 x 10^6^/mL in EZT buffer: HBSS+/+ containing 20 mM HEPES, and 0.1% human serum albumin (HSA, Grifols Biologicals, Los Angeles, CA), and EZ-TAXIScan™ (Effector Cell Institute, Tokyo, Japan) movies were obtained as previously described (36, 37). In brief, migration chambers containing 6-channel chips were assembled. Control and infected neutrophils were washed, and 10µL of each cell suspension was added into the top chamber. Chemokine concentration gradients were formed by injecting 1 µL of either 10 nM recombinant human C5a (Thermo Fisher Scientific),1 µM fMLF (Santa Cruz Biotechnology, Dallas, TX), or 5 nM recombinant human IL-8 (Millipore Sigma) into the bottom chamber of each channel, with injection of buffer only used as the negative control. Movies were recorded with 1 sec time lapse intervals between channels for a total of 60 min (3 images/channel/min). Where indicated, PMNs were treated at time zero with 25 µM Blebbistatin or 15 µM Y-27632 (both from Abcam, Cambridge, MA). For analysis, migrating cells were manually tracked using ImageJ software, and the percentage of motile cells for each condition was calculated. Chemotactic indices (CI) were calculated as the ratio of net path length toward the bottom well relative to the total path length. Additionally, average instantaneous velocities (IV, µm/min) were calculated with *xy*-coordinates scaled to 1μm and set time of video to 20 sec. In each case, at least 10 motile cells/condition were tracked per experiment.

#### Under-agarose assay

Neutrophils were suspended in HBSS-/- at 1 x 10^6^/mL and under-agarose chemotaxis assays were performed as previously described (38). In brief, sterile, acid-washed coverslips were placed in the center of 35 mm dishes and each dish was filled with 2 mL of 3% ultra-pure agarose (Invitrogen, Eugene, OR). Two 3.5mm diameter wells spaced 2.2 mm apart were cut in the solidified agarose over the coverslip. Approximately 10,000 neutrophils in 10µL were pipetted into one well and 10μL of fMLF (1μM final concentration) was added to the other well, and dishes were incubated at 37°C for 30 min. Next, agar was removed from each dish and neutrophils on each coverslip were fixed in 10% formalin for 15 min at room temperature, washed with HBSS-/- and stained with Hema-3 reagents (Fisher Scientific, Waltham, MA) as we described (29, 39). Images were acquired using a Leica DMi8 light microscope light microscope and 63x objective (Leica Microsystems, Inc., Deerfield, IL).

#### MatTek™ migration assay

Neutrophils were washed twice and resuspended at 1 x 10^6^/mL in Hepes-buffered RPM-1640 supplemented with 10% HI-FBS, plated on the coverslip in the center of each MatTek™ 35 mm dish (MatTek Corporation Ashland, MA), and allowed to attach for 30 min at 37°C under microaerophilic conditions. After adhesion, 10 µL of 10 µM fMLF was added from a point-source near the edge of the dish. After 30 min at 37°C, neutrophils were fixed and processed for fluorescence microscopy as described below.

#### Tapered channel microfluidics assay

Tapered channel microfluidics devices were fabricated in the BioMEMS Resource Center of the Massachusetts General Hospital and bonded to glass-bottom 6-well MatTek™ dishes as previously described (40). Each device contained 500 μm-long channels in groups of three, each with a cross-sectional area of 20 μm^2^ at the cell loading chamber end that tapered to 3-6 μm^2^ at the chemoattractant chamber. Device set up began with establishment of a chemical gradient that increased toward the chemoattractant chamber. Five microliters of 1μM fMLF was pipetted into each device through the inlet and each device was topped off with medium before being placed in a desiccator vacuum for 15 min. The negative pressure allowed fMLF to flow throughout one end of the device. Four milliliters of medium was then used to cover the device in the 6-well plate, and each device was washed with 10 μl of medium. For this assay, PMNs were stained with 0.5 µg/mL Hoechst 33342 (Fisher Scientific) for 5 min at 37°C and washed twice before 3 μl of neutrophils at 20 x10^6^/mL were pipetted into the device inlet. Time-lapse images of neutrophil migration were captured over 2 hours using an Olympus IX-81 inverted microscope equipped with a 20x phase contrast objective and a 37°C, 10% CO_2_ environmental chamber in the University of Iowa Central Microscopy Research Facility.

### Flow cytometry

Neutrophils were fixed in 1% paraformaldehyde (PFA), washed and blocked in HBSS (+/+) containing 4% non-fat dry milk and 4% HI-FBS and then stained with FITC-conjugated anti-CXCR1 mAb at 1:25x (5A12, BD Biosciences), APC-conjugated anti-CXCR2 mAb diluted 1:5x (6C6, BD Biosciences), Alexa Fluor (AF) 647-conjugated anti-FPR mAb diluted 1:20x (5F1, BD Biosciences) and/or FITC-conjugated anti-CD88 mAb diluted 1:25x (S5/1, BioLegend, San Diego, CA). All mAbs were diluted in blocking buffer. After incubation on ice for 60 min, cells were washed, resuspended in cold PBS and analyzed using an Accuri C6 Plus flow cytometer (BD Biosciences). At least 10,000 events/sample were acquired. Data were analyzed with FlowJo V10 software.

### Confocal microscopy

Our standard fixation conditions (41) were used for detection of RhoA, Myosin IIA and ROCK II. In brief, neutrophils in MatTek™ dishes were fixed in 10% formalin (Sigma-Aldrich, St. Louis, MO) for 15 min at room temperature and then permeabilized for 5 min in 1:1 cold methanol-acetone. After rinsing in PBS supplemented with 0.5 mg/mL NaN_3_ and 5 mg/mL BSA (PAB), cells were incubated at 4°C overnight blocking buffer (PAB+10% horse serum). To detect F-actin cells were fixed and permeabilized simultaneously in actin-fixation buffer (0.1% glutaraldehyde, 3.7% PFA and 0.15 mg/mL saponin) as previously described by DesMarais et al. (42), and then blocked overnight as described above. To detect microtubules (MTs) cells were fixed for 15 min in 2% PFA/0.1% glutaraldehyde in and then permeabilized with 0.5% SDS as we recently described (35) prior to blocking as described above.

Blocked cells were stained for 1 hour at room temperature with a 1:250 dilution of mouse anti-RhoA mAb (ab54835, abcam), 1:500 dilution of rabbit anti-Myosin IIA pAb (ab75590, abcam), 1:250 dilution of rabbit anti-ROCK II pAb (ab66320, abcam), 1:40 dilution of rhodamine-conjugated phalloidin (Invitrogen), 1:500 dilution of rabbit anti-α-tubulin (EP1332Y, abcam) and/or 1:500 dilution of mouse mAb specific for Phospho-Myosin Light Chain 2 Ser19 (#3675, Cell Signaling Technology, Danvers, MA). Affinity-purified F(ab’)_2_ secondary antibodies conjugated to AF488 or Dylight 549 were from Jackson ImmunoResearch Laboratories (West Grove, PA) and were used at 1:200 dilution. To detect active CD11b, FITC-conjugated mouse mAb clone CBRM1/5 (NB100-77754, Novus Biologicals, Centennial, CO) was utilized at 1:25 dilution. Primary and secondary antibodies were diluted in blocking buffer. Coverslips were mounted to MatTek™ dishes using ProLong Diamond plus DAPI (Thermo Fisher Scientific) and cells were analyzed using a Leica Stellaris 5 confocal microscope equipped with a 63x objective (Leica Microsystems Inc.). Z-stack images were deconvoluted using Leica Lightning software. 3D constructions and processing were conducted using Oxford Instruments Bitplane Imaris Software version 9.2.1 (Santa Barbara, CA). At least 100 cells were analyzed per condition and donor. All images shown are representative of at least three independent determinations.

### SDS-PAGE and immunoblotting

At 4 and 8 h after isolation or infection with *H. pylori*, neutrophils were left in medium alone or treated with 15 μM Y-27632 or 25 μM Blebbistatin for 30 min and then stimulated for 5 min with 100nM fMLF. Immediately thereafter, cells were pelleted, washed and resuspended in ice-cold PBS containing protease and phosphatase inhibitors and then lysed by addition of NP-40 to 1% final concentration as we previously described (43). Total protein in each lysate was quantified using the Pierce BCA assay (Thermo Fisher Scientific) prior to boiling in SDS-PAGE sample buffer. Proteins were separated on NuPAGE 10% Bis-Tris protein gels (Invitrogen) and then transferred to polyvinylidene difluoride membranes using Trans-Blot Turbo Transfer packs in the Trans-Blot Turbo system (both from Bio-Rad, Hercules, CA). Membranes were blocked in TBST containing 5% BSA and probed with 1:1,000 dilution of mouse mAb specific for Phospho-Myosin Light Chain 2 Ser19 (#3675, Cell Signaling Technology) prior to stripping and reprobing with 1:2,000 dilution of mouse mAb specific for GAPDH (clone 6C5, Calbiochem, San Diego, CA) as a loading control. Horseradish peroxidase-conjugated secondary antibodies (GE Healthcare, Chicago, IL) were used at 1:2,000 dilution. Bands were detected using Pierce SuperSignal™ West Pico PLUS reagents (Thermo Fischer Scientific) and a LI-COR Odyssey imaging system (LI-COR Biosciences, Lincoln, NE). Data were analyzed using ImageJ software.

### Cell adhesion assays

Neutrophils were suspended in Hepes-buffered RPMI-1640 supplemented with 10% HI-FBS at 1 x 10^6^/mL and plated on acid-washed coverslips that were precoated with 0.1 mg/mL fibrinogen (F-8630, Sigma-Aldrich) or autologous human serum in 24 well plates and then stimulated with 100 nM fMLF for 5 min. Thereafter, the medium was aspirated, and coverslips were washed twice with medium prior to fixation and Hema-3 staining. The number of adherent cells was quantified by manual counting of 10 separate random fields of view per sample and condition using a Leica DMi8 light microscope equipped with a 40x objective (Leica Microsystems Inc.).

### Quantitation of phagocytosis and phagocytic killing

Yeast zymosan particles (Sigma-Aldrich) and *Staphylococcus aureus* USA300 LAC (obtained from Dr. William Nauseef, University of Iowa) were opsonized with 50% normal human serum and used for synchronized phagocytosis assays as we previously described (32, 33, 41). Opsonized zymosan (OpZ) was used at an MOI of 4:1 and *S. aureus* was used at an MOI of 5:1-7:1. After incubation at 37°C for 15-20 min, cells were fixed and stained with Hema-3 reagents. Phagocytosis was quantified using light microscopy, and in each case both the percentage of neutrophils that ingested OpZ or *S. aureus* and the number of particles per infected cell were scored. In each case, at least 100 cells/condition/experiment were analyzed. The ability of neutrophils to kill ingested *S. aureus* (MOI 5:1) was quantified at 2 hours by enumeration of intracellular colony forming units released by saponin lysis as we described (33).

### Detection of cytosolic calcium

Neutrophils were suspended at 1x10^6^/mL in phenol red-free Hepes-buffered RPMI-1640 medium supplemented with L-glutamine and 10% HI-FBS and incubated under microaerophilic conditions in the presence and absence of *H. pylori* for 4 or 8 hours. Thereafter, cells were centrifuged and resuspended in HBSS +/+ containing 5µM final concentration of the fluorescent calcium indicator Fluo3-acetoxymethyl ester (Fluo3-AM, Invitrogen) and incubated for 30 min at room temperature in the dark. After two washes with HBSS+/+ to remove excess Fluo3-AM, 1x10^5^ neutrophils were loaded into duplicate wells of black, clear-bottom 96-well plates and placed in a CLARIOstar^PLUS^ microplate reader (BMG Labtech, Cary, NC) set for fluorescence excitation at 485 nm and emission at 538 nm. Basal calcium levels were monitored for 30 sec prior to injection of 1µM fMLF. Relative stimulus-induced changes in cytosolic calcium were tracked by measurements of fluorescence over the next 200 sec.

### Statistical analysis

Unless indicated otherwise, for experiments with one control and one experimental group, data were analyzed by paired Student’s *t*-test. To compare all groups to one another, we used two-way ANOVA followed by Tukey’s multiple comparisons post-test. All analyses utilized GraphPad Prism version 8 or version 9 software. Experiments were performed at least three times. In each case, *p*< 0.05 was considered statistically significant. Details are provided in the Figure legends.

## Results

### *H. pylori*-infected neutrophils polarize normally but are defective for chemotaxis due to defects in uropod contraction and detachment

**Figure 1:**
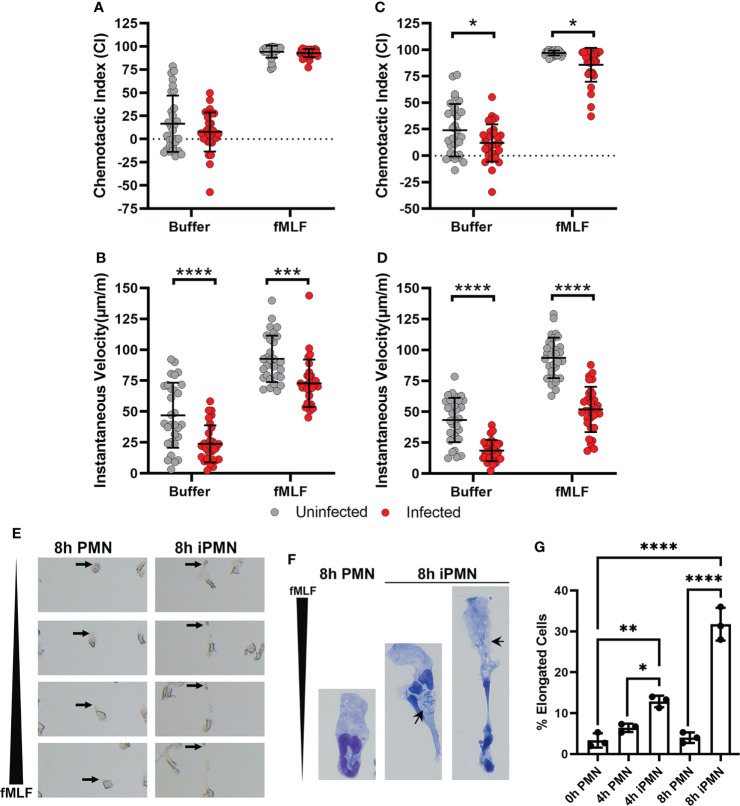
Chemotaxis of *H*. *pylori*-infected neutrophils to fMLF is impaired. **(A–E)** PMNs were left untreated or infected with *H*. *pylori* for 4 **(A, B)** or 8 hours **(C–E)** at 37°C prior to analysis of cell migration toward fMLF or buffer using an EZ-TAXIScan™ live cell imaging system. Single cell tracking was used to determine chemotactic indices **(A, C)** and instantaneous velocities (μm/min) **(B, D)**. Graphs show the mean ± SD from three independent experiments. Symbols are data for individual cells. Statistical significance was determined by two-way ANOVA and Tukey’s multiple comparison’s post-test: *p<0.05,***p<0.001, ****p<0.0001, as indicated. **(E)** Still images from EZ-TAXIScan™ videos show the morphology of representative control and infected cells migrating toward fMLF at 8 hours after isolation or initiation of infection. Arrows mark the trailing uropod of each cell. **(F)** Hema-3 staining shows the morphology of control and infected cells that migrated toward fMLF in the under-agarose assay. Arrows point to bacteria in infected cells. **(G)** Pooled under-agarose assay data indicate the percentage of elongated cells at each assayed time point as the mean ± SD from three independent experiments. Statistical significance was determined by two-way ANOVA and Tukey’s multiple comparisons post-test. *p<0.05, **p<0.01, ****p<0.0001, as indicated. iPMN, infected PMNs. Long black wedges indicate the fMLF concentration gradient in panels **(E, F)**.

To determine the potential influence of *H. pylori* infection on neutrophil migration we utilized first the EZ-TAXIScan™ complete live cell imaging system (36, 37) and directly compared migration of infected and uninfected PMNs to fMLF with buffer used as the negative control. As our original objective was to elucidate possible effects of hypersegmentation on PMN migration, and this morphological change is first apparent in a minority of PMNs at 6 hours post infection (hpi) (29), we performed our initial experiments at 4 hours to establish an initial baseline. As shown in **Figure 1A**, chemotactic indices (CIs) obtained for uninfected and infected PMNs were similar and in both cases migration toward fMLF was enhanced relative to the buffer control. However, the data also revealed a defect in migration speed, as the instantaneous velocity (IV) of the *H. pylori*-infected cells was significantly lower than their uninfected counterparts (**Figure 1B**). By 8 hours, differences between the infected and uninfected PMNs were more pronounced, as indicated by significant defects in directionality of cell migration as well as speed (**Figures 1C, D**).

The molecular events that underlie rapid, amoeboid migration of neutrophils have been extensively studied (3, 9, 10, 16, 44, 45). Fundamental to this process is initial chemoattractant gradient sensing by surface G-protein-coupled receptors (GPCRs). Gradient sensing leads to cell polarization that is characterized by a broad, often ruffled, leading lamellipodium and a narrower cell rear. Rapid net forward movement is driven by contraction of the cell rear which propels the cell body forward that is coupled to detachment of the contracted tail to achieve net forward cell movement. Careful examination of the EZ-TAXIScan™ videos (**Supplementary Videos 1–4**) demonstrated that infected PMNs were able to polarize in the direction of the gradient and extended their leading edges. However, many of these cells became highly elongated as they migrated toward fMLF at both assayed time points, a defect that was attributed to impaired uropod retraction, which was also apparent in the still images from the videos that are shown in **Figure 1E**.

We confirmed these data by analysis of cells that migrated toward fMLF in the under-agarose assay (**schematic in** **Supplementary Figure 1**). Representative images of Hema-3-stained cells each taken at the same magnification illustrate the elongated morphology of the migrating, infected PMNs (**Figure 1F**). Small black arrows in these images show that bacteria are randomly distributed and can be present behind or in front of the nucleus. The rightmost image also shows that the contracted cell rear can ensnare some or all of the nuclear lobes. In agreement with prior studies, we defined elongation as cells ≥250% the length of uninfected controls (16). Quantitation demonstrated that the percentage of elongated infected cells was significantly increased by 4 hpi and was more pronounced by 8 hpi (**Figure 1G**). In contrast, prolonged incubation *in vitro* did not elicit this behavior in uninfected control PMNs placed in a chemotactic gradient. Taken together, these data demonstrate that *H. pylori* infection progressively impaired neutrophil chemotaxis toward fMLF as indicated by defects in cell directionality and speed that correlated with cell elongation and a uropod retraction defect.

**Figure 2:**
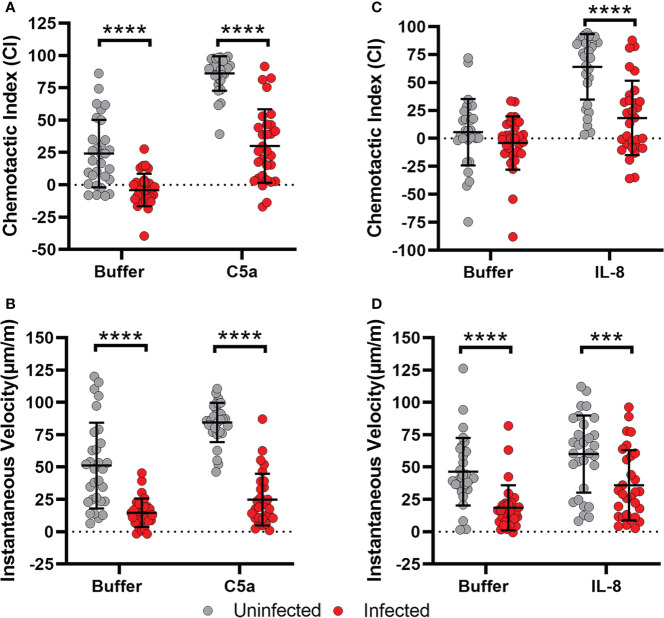
Migration of infected cells toward C5a and IL-8 is also impaired. **(A, B)** Chemotactic indices and instantaneous velocities of control and *H*. *pylori*-infected PMNs migrating in a C5a gradient. **(C, D)** Chemotactic indices and instantaneous velocities (μm/min) of control and infected PMNs migrating in an IL-8 gradient. Graphs indicate the mean ± SD from three independent experiments. Symbols show individual cell data. Statistical significance was determined by two-way ANOVA and Tukey’s multiple comparisons post-test. ***p<0.001, ****p<0.0001, as indicated.

The chemotaxis defect described here was not specific for fMLF, as similar results were obtained in assays of PMN migration toward C5a and IL-8 as indicated by EZ-TAXIScan™ video analysis (**Figure 2**). Moreover, the surface abundance of the formyl peptide receptor (FPR), the C5a receptor CD88, and the IL-8 receptors CXCR1 and CXCR2 were similar on control and infected cells at all time points analyzed over 8 hours of incubation at 37°C (**Supplementary Figure 2**), results that are in keeping with the ability of both control and infected PMNs to acquire a polarized morphology by sensing attractants in their milieu. Based on these data, we conclude that *H. pylori*-infected neutrophils demonstrated progressively impaired chemotaxis to both intermediate (IL-8) and end-target chemoattractants (C5a, fMLF) that was apparent by 4 hpi, and as such preceded hypersegmentation.

### F-actin structures and localization of RhoA, ROCKII and myosin IIA are disrupted by *H. pylori*

Regulated actin polymerization is critical for neutrophil chemotaxis such that Rac1 activation downstream of GPRCs drives front-to-back polarization and generation of an F-actin-rich lamellipodium at the leading edge, whereas active RhoA and non-muscle myosin IIA localize to the cell rear and provide the contractile force required to retract the cell rear and propel the cell body forward. Thus, rapid amoeboid migration is defined by distinct zones of frontness and backness (9).

**Figure 3:**
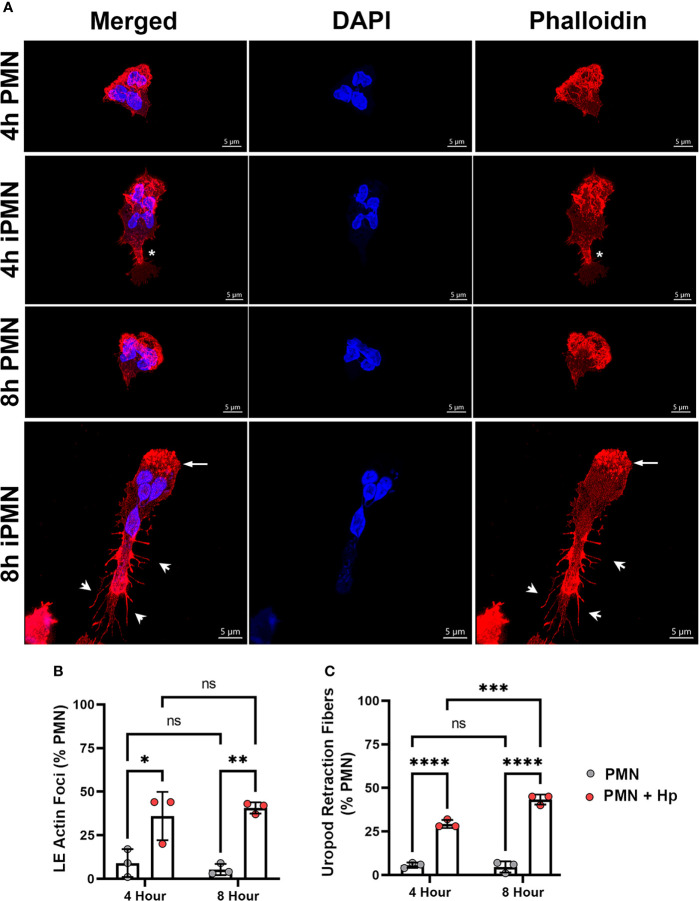
Infected PMNs display progressively modified actin localization during migration. **(A)** Images are 3D confocal Z-stacks of control and infected cells migrating toward fMLF in MatTek™ dishes assayed at 4 and 8 hours. Fixed cells were double-stained to detect DNA (DAPI, blue) and F-actin (rhodamine-phalloidin, red). *Asterisks* indicate an area of F-actin enrichment behind the nucleus of a 4 hour iPMN. *Arrows* and *arrowheads* indicate actin foci at the leading edge and actin-rich spikes/retraction fibers decorating the elongated uropod of an 8 hour iPMN, respectively. Images are representative of three independent experiments. Scale bar = 5 μm. iPMN, infected PMN. **(B, C)** Pooled confocal data indicate the percentage of uninfected and *H*. *pylori*-infected PMNs that exhibited enhanced leading edge (LE) F-actin foci **(B)**, F-actin-rich retraction fibers at the rear uropod **(C)**. At least 100 cells/experiment/condition were scored. Data are the mean ± SD from three independent experiments. Data were analyzed by two-way ANOVA and Tukey’s multiple comparisons post-test. *p<0.05, **p<0.01, ***p<0.001, ****p<0.0001, ns, not significant, as indicated. Hp, *H*. *pylori*.

The aberrant morphology and impaired chemotaxis of *H. pylori*-infected cells suggested that actin dynamics may be impaired. To gain insight into the nature of the possible underlying defects, control and infected cells were plated on serum-coated glass coverslips in MatTek™ dishes and stimulated with a point source of fMLF to trigger polarization and directed migration. Thereafter, cells were fixed, stained, and analyzed using confocal microscopy. In our first series of experiments, cells were stained with rhodamine-phalloidin to detect F-actin along with DAPI to detect DNA. The data in **Figure 3A** demonstrate that control PMNs migrating toward fMLF exhibited a classical polarized (fan-shaped) morphology at both assayed time points. In keeping with this, actin ruffles were present throughout the wide leading lamellipodium, but F-actin was absent or nearly absent in the contracted uropod (**Figure 3A**). Ruffled lamellipodia were also present at the leading edges of *H. pylori*-infected cells assayed at 4 hpi, but actin was not absent from the cell rear. Rather, regions of F-actin enrichment were apparent behind the nucleus, creating an area of constriction within the elongating trailing edge (**Figure 3A**, *asterisk*). By 8 hpi changes in F-actin were more pronounced, as leading lamellipodia contained numerous actin foci rather than ruffles (**Figure 3A**, *arrows*) and trailing uropods were stained intensely with rhodamine-phalloidin and were notable for elaboration of numerous F-actin-rich projections or spikes at the sides and rear that resembled retraction fibers (46) (**Figure 3A**, *arrowheads*). Quantitation demonstrated that both anterior actin foci and trailing retraction fibers were significantly increased by *H. pylori* infection (**Figures 3B, C**).

**Figure 4:**
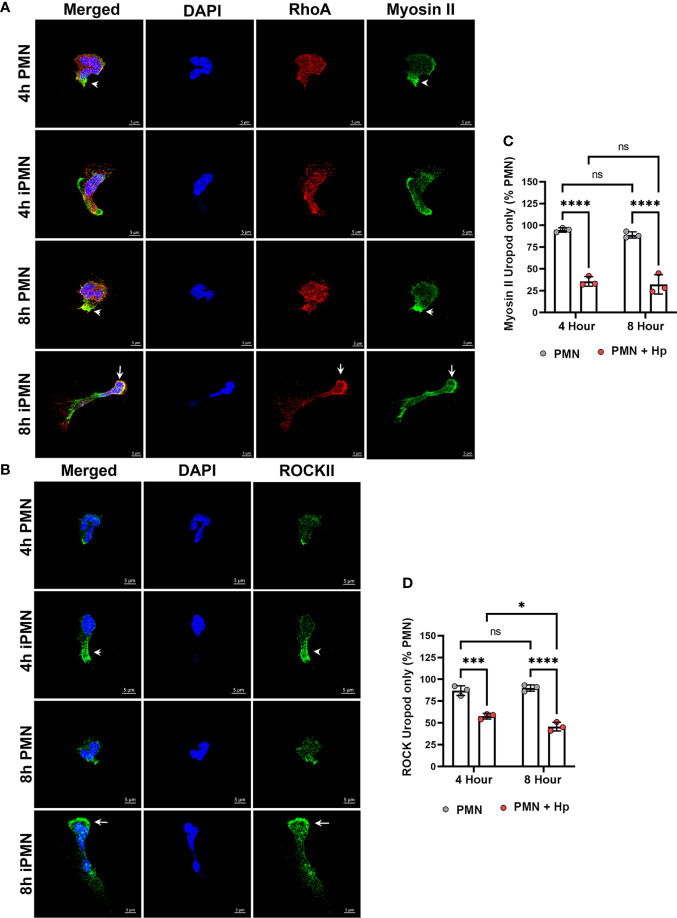
RhoA, Myosin II and ROCK II are mislocalized in migrating, infected neutrophils. Cells that migrated toward fMLF in MatTek™ dishes at 4 or 8 hours were fixed and processed for confocal microscopy. **(A)** Z-stack images of control and infected PMNs (iPMNs) (were stained to detect DNA (DAPI, blue), RhoA (red) and myosin IIA (green). *Arrowheads* indicate myosin II enrichment at the uropod of control PMNs. *Arrows* indicate accumulation of RhoA and myosin IIA at the front of 8 hour iPMNs. **(B)** Z-stack images of control and infected PMNs were stained to detect DNA (DAPI, blue) and ROCK II (green). *Arrowheads* indicate ROCK II accumulation in the elongated uropod of a 4 hour infected PMN (iPMN). *Arrows* indicate ROCK II accumulation at the leading edge of an 8 hour iPMN. Images shown are representative of three independent experiments. Scale bar = 5 μm. **(C, D)** Pooled confocal data indicate the percentage of uninfected and *H*. *pylori*-infected PMNs that exhibited myosin II staining confined to the uropod **(C)** or ROCK II staining confined to the uropod **(D)**. At least 100 cells/experiment/condition were scored. Data are the mean ± SD from three independent experiments. Data were analyzed by two-way ANOVA and Tukey’s multiple comparisons post-test. *p<0.05, ***p<0.001, ****p<0.0001, ns, not significant, as indicated. Hp, *H*. *pylori*.

As noted above, RhoA defines PMN backness and signaling from RhoA to myosin IIA mediates uropod detachment and retraction *via* actomyosin contraction. In our next series of experiments, we determined if *H. pylori* altered localization of these important uropod components. At both assayed time points, RhoA was diffusely distributed with slight enrichment at the cell rear whereas myosin IIA was highly enriched at the uropod and occasionally at the leading edge of control neutrophils (**Figure 4A**), in agreement with published data (10, 14, 16, 47). Both RhoA and myosin IIA were present throughout the uropods of PMNs infected with *H. pylori* for 4 hours but at later times this was not the case, as by 8 hpi RhoA was depleted from the cell rear and enriched at the leading edge (**Figure 4A**, *arrows*). On the other hand, myosin IIA was apparent throughout infected cells, including trailing retraction fibers, in addition to its enrichment at the cell front where it colocalized with RhoA (**Figure 4A**, *arrows*). ROCKII links RhoA to myosin IIA activation, and in keeping with this ROCKII was present throughout polarized control PMNs with slight enrichment at the uropod (**Figure 4B**). Uropod enrichment of ROCK II was more readily apparent 4 hours after *H. pylori* infection (**Figure 4B**, *arrowhead*). However, by 8 hpi ROCK II was mislocalized, and like RhoA was enriched at the leading edge yet depleted from the cell body and rear (**Figure 4B**, *arrow*). Quantitation of myosin IIA and ROCKII localization are shown in **Figures 4C, D**, respectively.

### Effects of Blebbistatin and Y-27632 are similar but not identical to *H. pylori*

**Figure 5:**
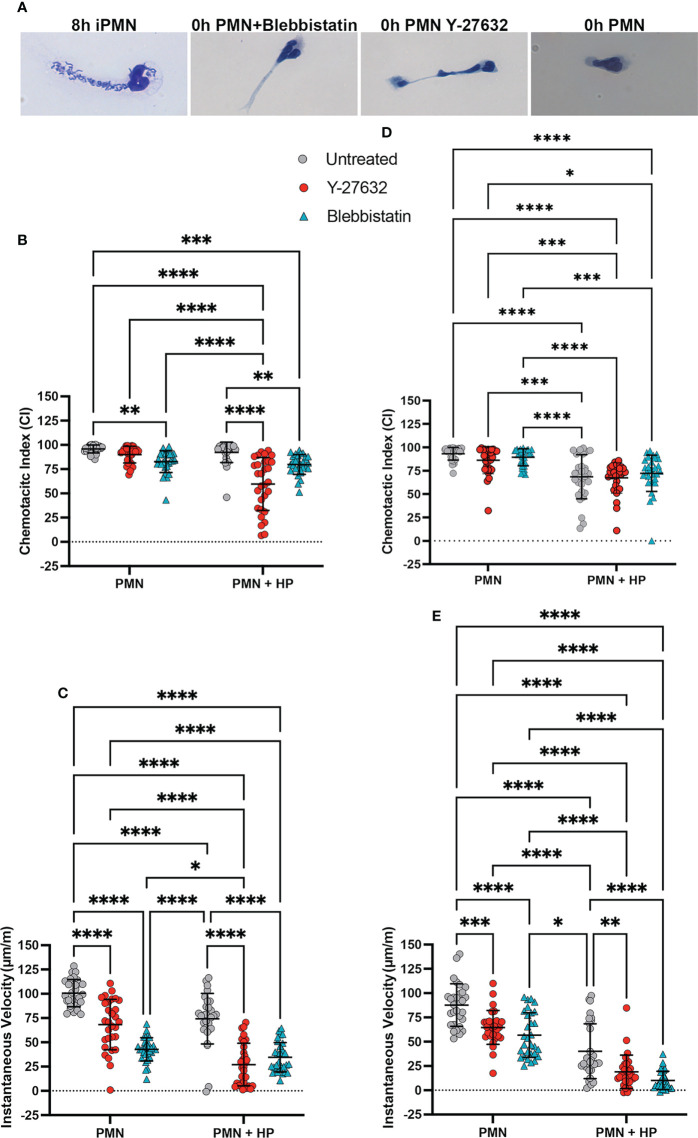
Effects of Blebbistatin and Y-27632 on chemotaxis with and without (*H*) *pylori* infection. **(A)** Representative images shown Hema-3 stained 8 hour infected PMNs (iPMNs), 0 hour control PMNs, and control PMNs treated with Blebbistatin or Y-27632 migrating toward fMLF in the under-agarose assay. **(B–E)** EZ-TAXIScan™ data obtained at 4 hours **(B, C)** and 8 hours **(D, E)**. Chemotactic indices **(B, D)** and instantaneous velocities (μm/min) **(C, E)** are shown for untreated control PMNs and cells treated with Blebbistatin or Y-27632 with and without prior *H*. *pylori* infection, as indicated. Graphs show the mean ± SD (n=3). Symbols are data for individual cells. Statistical significance was determined by two-way ANOVA and Tukey’s multiple comparisons post-test. *p<0.05, **p<0.01,**p<0.01, ***p<0.001, ****p<0.0001 for the indicated comparisons.

Uropod retraction defects can be induced in neutrophils by treatment with the myosin IIA inhibitor Blebbistatin or the ROCKII inhibitor Y-27632 (15). We compared the effects of these drugs to *H. pylori* infection by analysis of cell morphology, chemotaxis and protein localization. Hema-3 staining of neutrophils migrating toward fMLF in the under-agarose assay showed that freshly isolated cells polarized normally but were highly elongated after treatment with Blebbistatin or Y-27632, and as such resembled cells that had been infected with *H. pylori* for 8 hours (compare **Figures 5A–1F**). Both drugs significantly impaired neutrophil directionality and speed as judged by EZ-TAXIScan™ imaging at 4 and 8 hours, and this was exacerbated when combined with infection, particularly with respect to IV (**Figures 5B–E** and **Supplementary Videos 5–12**).

**Figure 6:**
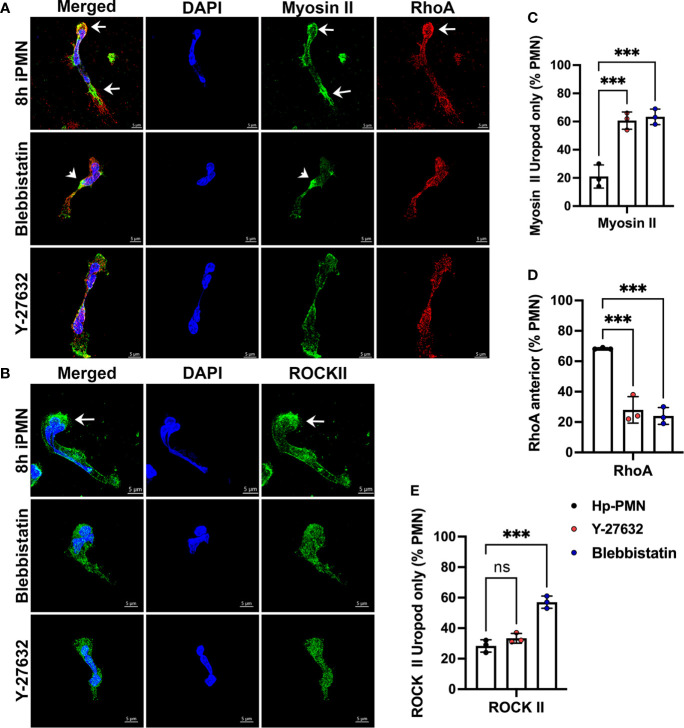
Effects of Blebbistatin and Y-27632 on localization of Myosin IIA, RhoA, and ROCK II. Freshly isolated neutrophils in MatTek™ dishes were treated with 25 μM Blebbistatin or 15 μM Y-27632 and allowed to migrate toward fMLF. Cells infected with *H*. *pylori* for 8 hours are shown for comparison. Fixed cells were stained to detect DNA (DAPI, blue), myosin IIA (green) and RhoA (red) **(A)**, or DNA (DAPI, blue) and ROCK II (green) **(B)**. *Arrows* indicate protein enrichment at the front or rear of infected PMNs. *Arrowheads* indicate enrichment of myosin IIA directly behind the nucleus of cells treated with Blebbistatin. 3D confocal Z-stack reconstructions shown are representative of three independent experiments. Scale bar = 5 μm. **(C–E)** Pooled data indicate the percentage of *H. pylori*-infected, Y27632- or Blebbistatin-treated neutrophils at 8 hours with uropod-restricted myosin IIA **(C)**, anterior accumulation of RhoA **(D)** or uropod-restricted ROCKII **(E)**. At least 100 cells per experiment and condition were scored. Data are the mean ± SD (n=3). Data were analyzed by one-way ANOVA and Tukey’s multiple comparisons post-test. As indicated, ***p<0.001, ns, not significant. Hp-PMN, *Helicobacter pylori*-infected PMN.

Confocal analysis showed that although both drug-treated and *H. pylori*-infected cells were elongated and defective for uropod retraction, the subcellular distributions of myosin IIA, RhoA and ROCKII differed as indicated by the representative images (**Figures 6A, B**) and pooled data (**Figures 6C–E**). All three treatments altered myosin IIA localization as compared with the uninfected controls (compare **Figures 4A, C**, **6A, C**). However, *H. pylori* disrupted myosin IIA localization to a greater extent than either inhibitor, as bipolar enrichment of myosin IIA was prominent following *H. pylori* infection, was weak or absent following Y-27632 treatment and was not observed in cells treated with Blebbistatin, which was instead associated with myosin IIA accumulation immediately behind the nuclear lobes (**Figure 6A**). At the same time, anterior accumulation of RhoA was specific to *H. pylori* infection (**Figures 6A, D**) and was no more apparent in drug-treated cells than the uninfected controls (16.7 ± 1.5%, n=3) (**Figure 4A** and data not shown). Finally, infection and Y-27632 caused greater delocalization of ROCK II than Blebbistatin, but only *H. pylori* elicited accumulation of this protein at the leading edge (**Figures 6B, E**), confirming results shown in **Figure 4B**.

**Figure 7:**
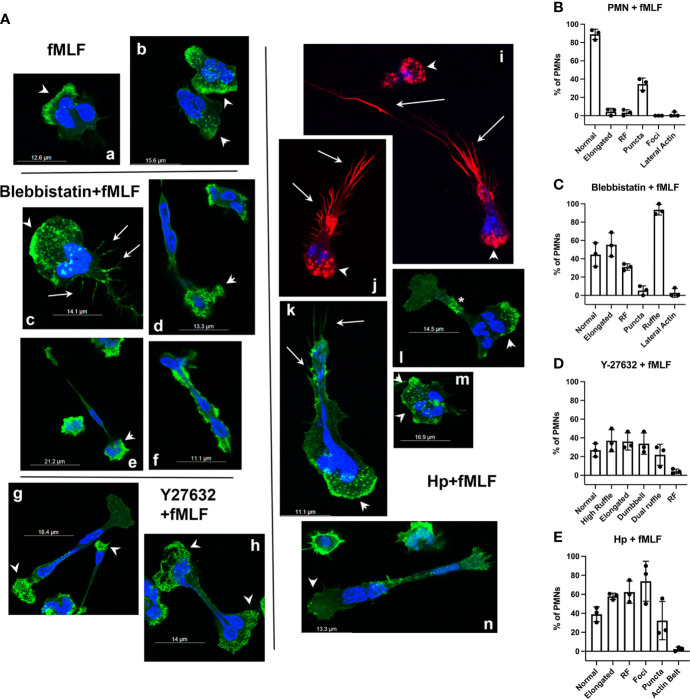
Effects of inhibitors and infection on F-actin structures in neutrophils migrating toward fMLF. **(A)** Representative confocal 3D Z-stack images of PMNs migrating toward fMLF in MatTek™ dishes are shown. Cells were stained with DAPI to detect DNA (blue) or rhodamine-phalloidin to detect F-actin (red or green pseudocolor). fMLF controls (**images a, b**). Blebbistatin-treated cells (**images c**–**f**). Y-27632-treated cells (**images g, h**). *H*. *pylori*-infected cells (**images i**–**n**). *Arrowheads*, actin-rich ruffles and foci at the leading edge or cell poles. *Arrows*, actin-rich spikes/retraction fibers at the uropod. *Asterisk*, F-actin belt. Hp, *H*. *pylori*. **(B–E)** Quantitation of F-actin features in control **(B)**, Blebbistatin-treated **(C)**, Y-27632-treated **(D)** or *H*. *pylori*-infected cells **(E)** treated with fMLF. Scored features include normal or elongated morphology, retraction fibers (RF), small actin puncta, large actin foci, lateral actin enrichment or actin belts as well as anterior ruffling of elongated, Blebbistatin-treated cells, elongated Y-27632-treated cells nearly all of which were dumbbell-shaped and dumbbell-shaped cells with ruffles at both ends (*Dual Ruffle*). Data are the mean ± SD from three independent experiments.

To further define these phenotypes, we extended our analysis to include cells stained with rhodamine-phalloidin (**Figure 7**). Control cells migrating toward fMLF in MatTek™ dishes were notable for accumulation of F-actin at leading edge and small actin puncta throughout the cytosol (**Figure 7A images a, b**, *arrowheads*). Following treatment with Blebbistatin, some cells exhibited wide lamellipodia and uropods that elaborated retraction fibers (**Figure 7A image c**, *arrowheads* and *arrows*, respectively), whereas other cells had actin ruffles at the narrowed leading edge (**Figure 7A images d, e**, *arrowheads*) and were highly elongated, leading to compression/trapping of one or more nuclear lobes. Rarely, cells with a tube-like morphology, intense lateral F-actin staining, and lack of clear anterior and rear ends were also observed (**Figure 7A image f**). Hallmarks of Y-27632-treated cells were elongated cells, almost all of which had a dumbbell morphology, with intense actin ruffling at one or both ends (**Figure 7A images g, h**) and nuclear lobes that appeared to be positioned randomly at one or both poles, or in the middle of elongated cells. Finally, cells infected with *H. pylori* for 8 hours were notable for the presence of large actin foci at the leading edge (**Figure 7A images i–n**, *arrowheads*) and elaboration of actin spikes/retraction fibers at the sides and rear of the elongated uropods, some of which trailed behind cells for long distances (**Figure 7A images i–k**, *arrows*). Other cells had a relatively normal morphology (**Figure 7A image m**) or were elongated but lacked trailing actin spikes (**Figure 7A image n**). Cells that were characterized by accumulation of an F-actin ‘belt’ rather than rear spikes (**Figure 7A image l**, *asterisk*) were more common at 4 hpi, as shown in **Figure 3A**. The morphological features associated with each treatment were quantified and are shown in **Figures 7B–E**. Considered together, these data indicate that actin rearrangements induced by *H. pylori* most closely resemble cells treated with Blebbistatin but differ with regard to actin morphology at the leading edge and the magnitude of trailing retraction fibers.

### *H. pylori* enhances phosphorylation of myosin IIA regulatory light chains

**Figure 8:**
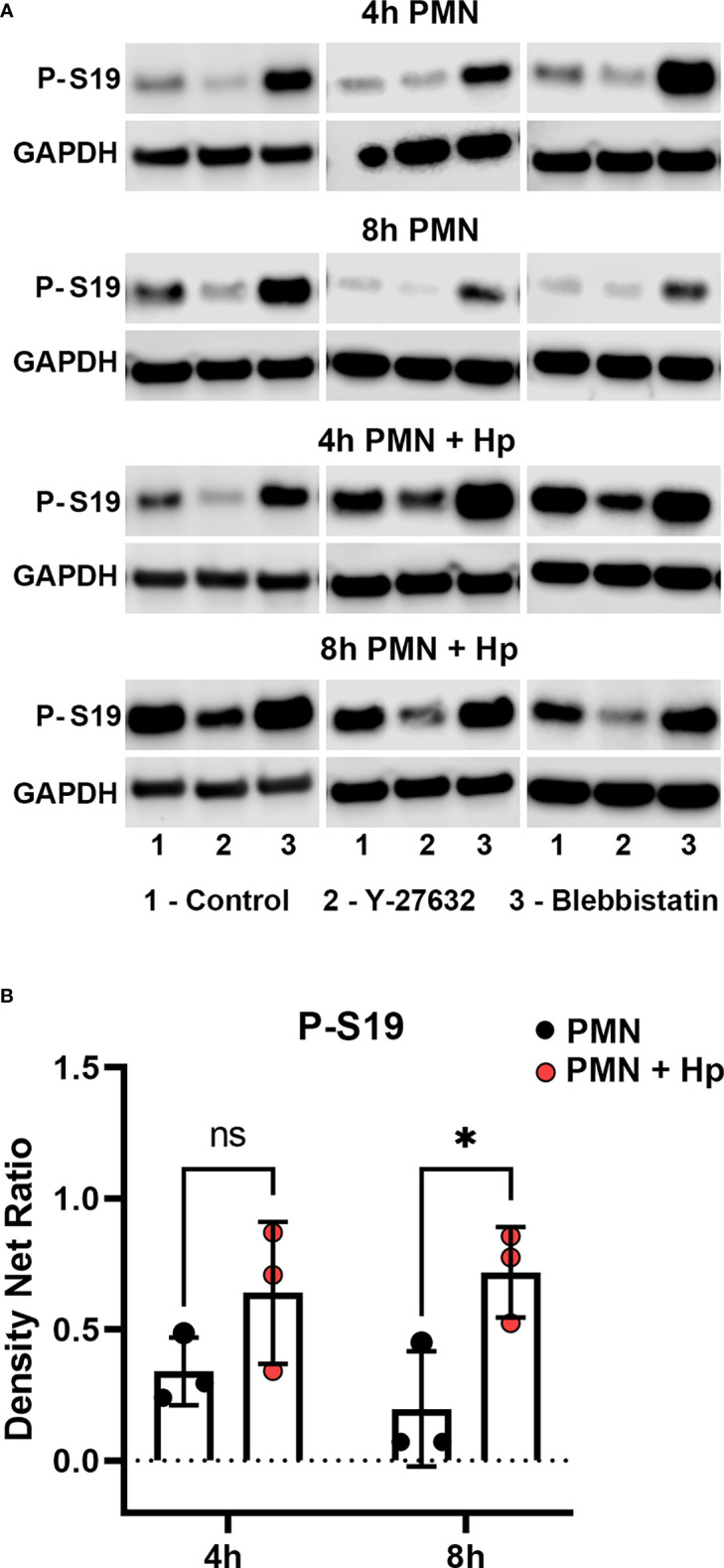
Effects of infection, Y-27632 and Blebbistatin on myosin IIA RLC S19 phosphorylation. Neutrophils were left untreated or infected with *H*. *pylori* for 4 or 8 hours in the presence and absence of Y-27632 or Blebbistatin, as indicated. Immunoblots of whole cell lysates were probed to detect myosin IIA RLC S19 phosphorylation prior to stripping and reprobing with antibodies to GAPDH as the loading control. **(A)** Data from three representative experiments. **(B)** Quantitation of normalized S19 phosphorylation intensity for uninfected and infected PMNs (in absence of drugs) at 4 and 8 h are the mean ± SD (n=3). ns, not significant. *p<0.05. Additional data analyses are shown in **Supplementary Figure 3**.

**Figure 9:**
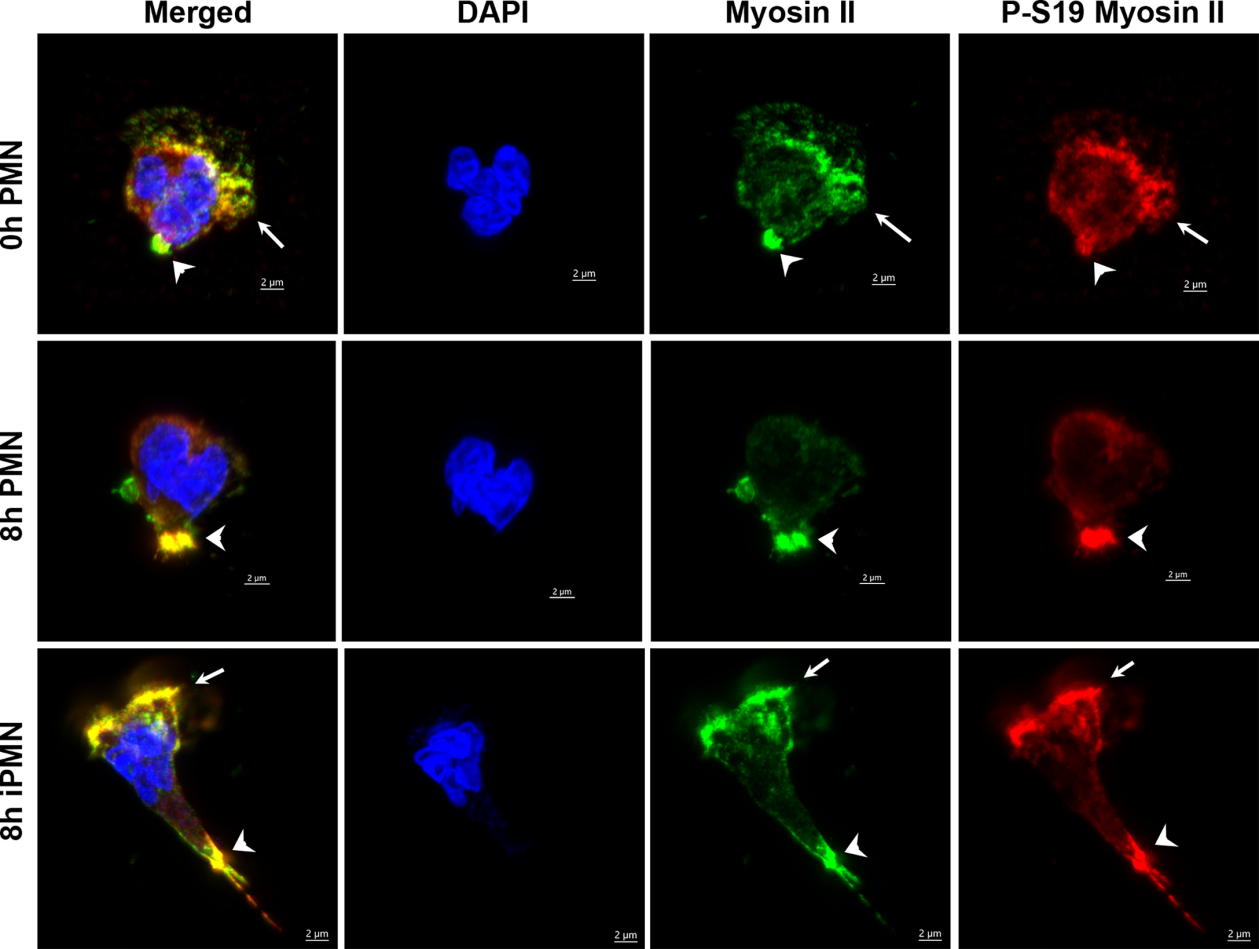
Subcellular localization of total and active myosin IIA phosphorylated on S19. Neutrophils were processed for confocal microscopy at 0 or 8 hours after isolation or 8 hours after infection with *H. pylori*. Representative Z-stack images were stained with DAPI to detect DNA (blue), myosin IIA (green) or S-19-phosphorylated myosin IIA (red). *Arrowheads* indicate colocalization and enrichment of total and S19-phosphorylated myosin IIA at the uropod. *Arrows* indicate colocalization and enrichment of total and S19-phosphorylated myosin IIA at the leading edge of cells infected with *H. pylori* (iPMN).

Activation of myosin IIA requires phosphorylation of its regulatory light chains (RLCs) on serine 19 (S19) (48). In PMNs, ROCKII activates myosin IIA directly by phosphorylating S19 and also favors its activity indirectly by phosphorylating and inhibiting its cognate phosphatase, both of which are inhibited by Y-27632 (10, 15, 49). In contrast, Blebbistatin directly inhibits myosin IIA ATPase activity at the rate-limiting step, acting distal to hydrolysis to prevent release of ADP and Pi (48, 50). Precisely how *H. pylori* inhibits uropod retraction is unknown. We now show by immunoblotting of cell lysates that RLC S19 phosphorylation in control PMNs migrating toward fMLF was low-moderate at both 4 and 8 hours. This signal was consistently enhanced by Blebbistatin and as expected was inhibited by Y-27632 (**Figure 8A** and **Supplementary Figure 3**). On the other hand, RLC S19 phosphorylation was slightly elevated at 4 hpi and significantly increased at 8 hpi with *H. pylori*, and although this signal remained sensitive to inhibition by Y-27632, the ability of Blebbistatin to further enhance this response was diminished (**Figures 8A, B** and **Supplementary Figure 3**). In addition, confocal analysis demonstrated that S19-phosphorylated RLCs in uninfected control PMNs were enriched at the uropod and in a subset of cells was also present at lower levels anterior to the nucleus of cells in fMLF gradients where it colocalized with myosin IIA heavy chains (**Figure 9**). In contrast, S19-phosphorylated and total myosin IIA were highly enriched at both poles of cells infected with *H. pylori* (**Figure 9**). Taken together, these data demonstrate that *H. pylori* does not disrupt chemotaxis by preventing myosin IIA phosphorylation.

### Microtubules orient normally toward the uropod after *H. pylori* infection

Resting PMNs contain relatively few MTs (35, 51). However, we demonstrated recently that MTs are more abundant and longer in *H. pylori-*infected neutrophils and play a key role in hypersegmentation *via* mechanotransduction across the nuclear membrane (35). It has also been shown that MTs increase in number during chemotaxis and in this case preferentially orient toward the uropod rather than radiating uniformly from the microtubule organizing center (MTOC) (52–55). The confocal images shown in **Supplementary Figure 4** confirm the effects of fMLF on MTs in control PMNs and show that the abundant MTs in *H. pylori*-infected cells were preferentially oriented toward the uropod of cells migrating toward fMLF regardless of whether these cells exhibit relatively normal polarization or a highly elongated morphology, as depicted in the 4 and 8 hpi images, respectively. These data indicate that MT orientation was not disrupted by *H. pylori* infection.

### Defective migration is specific for infection with live *H. pylori*

**Figure 10:**
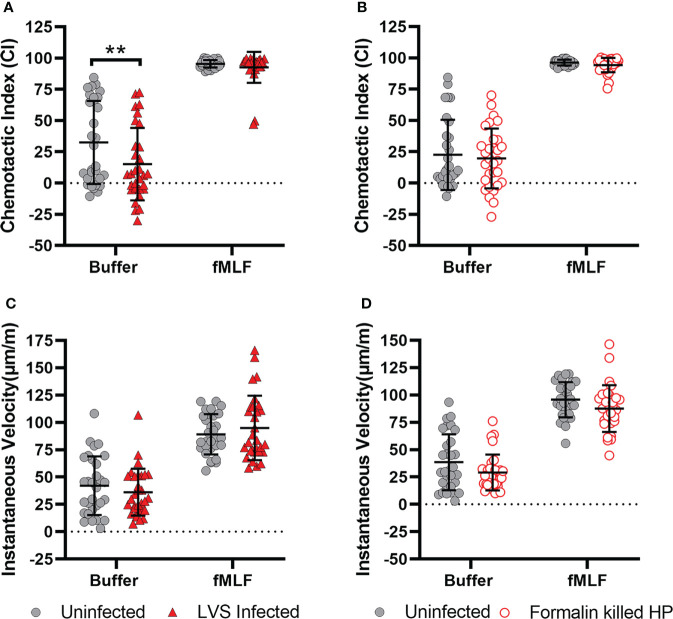
Inhibition of chemotaxis is specific for live *H. pylori* infection. Neutrophils were infected with *F. tularensis* strain LVS **(A, B)**, or formalin-killed *H. pylori* **(C, D)** and migration toward buffer and fMLF was assayed 8 hours later using EZ-TAXIScan™ imaging. Chemotactic indices **(A, C)** and instantaneous velocities (μm/min) **(B, D)** were calculated. Graphs display the mean ± SD for three independent experiments. Symbols are data for individual cells. Statistical significance was determined by two-way ANOVA and Tukey’s multiple comparison test. **p<0.01, as indicated. Other comparisons were not significant (*p*>0.05).

To determine if impaired chemotaxis was a general consequence of phagocytosis or bacterial infection, we utilized another bacterial pathogen, *Francisella tularensis.* Both *H. pylori* and *F. tularensis* evade killing by neutrophils and extend cell lifespan but unlike *H. pylori*, which persists inside phagosomes, *F. tularensis* escapes the phagosome and replicates in PMN cytosol (32, 39). Herein, we infected PMNs with *F. tularensis* strain LVS for 8 hours prior to EZ-TAXIScan™ imaging. When placed in an fMLF gradient, these cells were indistinguishable from the uninfected controls with respect to the directionality and speed of migration (**Figures 10A, B**). Nonetheless, the *F. tularensis*-infected cells exhibited a selective defect in directionality, but not speed, when migrating toward buffer alone. In additional experiments, we also analyzed PMNs 8 hours after uptake of formalin-killed *H. pylori* (**Figures 10C, D**). In this case, prior ingestion of killed bacteria had no discernable effect on neutrophil migration toward buffer or fMLF. These data suggest that impaired chemotaxis is not a general consequence of phagocytosis or infection.

The bacterial factors that disrupt chemotaxis of infected PMNs are unknown. As a first approach to addressing this question, we utilized our collection of isogenic mutants with deletions in genes encoding the *H. pylori* neutrophil activating protein (Δ*napA*), the VacA cytotoxin (Δ*vacA*), the type IV secretion system component CagE (Δ*cagE*), the secreted effector CagA (Δ*cagA*), or urease (Δ*ureAB*), an enzyme that is essential for bacterial survival in acidic environments and disrupts phagosome maturation in macrophages (18, 31). We demonstrated previously that all these strains can survive in PMNs for at least 24 hours and retain the ability to induce hypersegmentation and N1-like subtype differentiation (29). We now show by EZ-TAXIScan™ analysis that the mutant strains were also indistinguishable from wild-type *H. pylori* with respect to their ability to diminish cell directionality and speed during migration toward fMLF (**Supplementary Figure 5**).

### Infected cell migration in tapered 3D channels is not impaired

Neutrophils migrate in both 2D and 3D environments *in vivo* but the underlying mechanisms differ as 2D migration is integrin-dependent whereas 3D migration is not (9). We have shown that *H. pylori* infection disrupts PMN chemotaxis using EZ-TAXIScan™ imaging, the under-agarose assay and MatTek™ dishes, 2D migration systems that are relevant to *H. pylori*-neutrophil interactions at the surface of the gastric epithelium *in vivo* (20). To elucidate potential effects on integrin-independent migration, we utilized tapered channel microfluidic devices that circumferentially constrain PMNs migrating toward fMLF and recapitulate the integrin-independent migration that occurs in 3D systems (9, 56). In agreement with published data, live cell imaging identified four cell behaviors: persistent migration toward the chamber containing fMLF, stalled forward migration, oscillation in the channel and reverse migration (also called retrotaxis) (56). All four behaviors were exhibited by control and *H. pylori*-infected cells assayed at 4 and 8 hours (**Supplementary Figure 6**). In general, more cells were motile at 8 hours, but the overall behavior of control and infected cells was similar in two independent experiments. Taken together, our data suggest that *H. pylori* selectively impairs PMN migration in 2D environments.

### Adhesion of infected PMNs, subcellular localization of CD11b and cytosolic calcium are unchanged

As integrin-mediated adhesion is important for 2D migration and can amplify the effects of myosin-related inhibitors (9, 14, 52) we hypothesized that PMN adhesion may be enhanced. To test this, PMNs were plated on autologous serum-coated or fibrinogen-coated glass coverslips 8 hours after infection or isolation and stimulated with fMLF for 5 min. Subsequent light microscopy analysis suggested that infected cells bound somewhat more readily to coated surfaces than their uninfected counterparts, but these data were not statistically significant (*p*>0.06) (**Supplementary Figure 7A**).

In addition, CD11b accumulates in the uropod during chemotaxis and helps modulate myosin IIA contractility and cell adhesion (9). In agreement with this, CD11b localized to intracellular vesicles that were diffusely distributed in cells at rest and accumulated at the back of both control and infected PMNs in fMLF gradients (**Supplementary Figure 7B** and data not shown). Thus, impaired chemotaxis cannot be attributed to changes in cell adhesion or localization of CD11b/CD18. Similar data were obtained using antibodies specific for active CD11b (data not shown).

As dynamic changes in calcium are important for many aspects of neutrophil function and in some instances can influence myosin IIA RLC phosphorylation (14, 57), we utilized an established Fluo3-AM assay to determine relative cytosolic calcium levels of control and infected PMNs before and after fMLF stimulation (58). As shown in **Supplementary Figure 7C**, the data obtained at 4 and 8 hours for both sets of cells were nearly identical and did not differ significantly as judged by two-way ANOVA and Sidak’s multiple comparisons test.

### Neutrophil capacity for phagocytosis and killing are not impaired

Having established that *H. pylori* inhibits neutrophil chemotaxis, we determined if other fundamental aspects of cell function were impaired. To this end, we quantified neutrophil capacity to ingest serum-opsonized yeast zymosan particles (OpZ) as well as uptake and killing of opsonized *S. aureus*. To quantify phagocytic capacity, we determined both the percentage of neutrophils that contained OpZ or *S. aureus* (**Supplementary Figures 8A, C**) as well as the number of particles or staphylococci in each phagocytic cell (**Supplementary Figures 8B, D**). These data show that neutrophil phagocytic capacity was neither enhanced nor diminished by prior infection with *H. pylori*. Intracellular killing of *S. aureus* was also unchanged as indicated by measurement of recovered colony forming units (**Supplementary Figure 8E**).

## Discussion

The field of neutrophil plasticity is advancing rapidly and many PMN populations have been described. Nonetheless, our understanding of their shared and distinct properties is incomplete and relatively few studies have included an examination of chemotaxis. One recent study identified multiple chemotaxis defects in PMNs from severely ill ICU patients (59). Conversely, chemotaxis markers are upregulated in all neutrophil subsets on day 2 and day 3 post burn injury (60). In endotoxemia, a subpopulation of CD62L^low^/CD16^high^ PMNs arises that is defective for adhesion and migration to fMLF as compared to immature CD62L^high^/CD16^low^ banded cells but not relative to their mature CD62L^high^/CD16^high^ counterparts (61, 62). Results of another endotoxemia model suggest that LPS functions as a stop signal that arrests chemotaxis toward fMLF and IL-8 *via* extracellular ATP and P2Y1 signaling (63).

It is established that *H. pylori* elicits a chronic, neutrophil-dominant inflammatory response in humans that does not eliminate the infection and can progress from gastritis to ulceration or cancer. Infected neutrophils acquire an N1-like phenotype that is characterized by robust extracellular oxidant production, extended lifespan, profound nuclear hypersegmentation and a CD62L^low^/CD16^high^/CD11b^high^/CD66b^high^/CD63^high^ surface phenotype (18, 19, 21, 29). The results of this study advance understanding by demonstrating that *H. pylori*-infected PMNs also develop a progressive and significant defect in uropod detachment and retraction that impaired chemotaxis to fMLF and C5a as well as IL-8, and our combined use of live imaging and microscopy provided insight into the underlying mechanisms. EZ-TAXIScan™ videos show that infected cells polarized normally when placed in chemotactic gradients and established ruffled leading lamellae, results that are consistent with normal surface abundance of FPR, CD88, CXCR1 and CXCR2. As cells began to migrate, contraction of the cell rear pushed the cell body forward, but detachment and retraction of the uropod were impaired. Additional cycles of rear squeezing led to progressive cell elongation that frequently trapped one or more nuclear lobes within the constricted rear in parallel with narrowing of the leading lamellipodium.

A key feature of the self-organizing polarity of neutrophils is segregation of F-actin at the front and actomyosin bundles at the rear. Thus, F-actin is abundant in anterior lamellipodia and largely absent from the cell body and uropod (9, 10, 14). At the molecular level, the first apparent defect in *H. pylori*-infected neutrophils was delocalization of F-actin, as indicated by its accumulation at a zone of constriction behind the nucleus, in parallel with enrichment of myosin IIA, ROCKII and to a lesser extent RhoA throughout the distal, enlarging uropod at 4 hpi. By 8 hpi, infected cells were more highly elongated, and F-actin was abundant throughout enlarged uropods which also elaborated actin spikes and retraction fibers, and in many instances anterior ruffles were replaced by large actin foci. S19-phosphorylation of myosin IIA RLCs was significantly enhanced, and in marked contrast to uninfected cells phosphorylated and total myosin IIA were not confined to the uropod - as indicated by their simultaneous enrichment at the front and rear. At the same time, the subcellular distribution of ROCKII and RhoA was abnormal, as both proteins colocalized with myosin IIA in front of the nucleus and were depleted at the back. These data are significant as stable neutrophil polarity and rear contractility require the rearward concentration of RhoA, ROCKII and myosin IIA (11).

The effects of Blebbistatin on PMNs have been extensively studied and closely resemble the effects of *H. pylori* infection, suggesting that this pathogen may also inhibit myosin IIA activity to disrupt chemotaxis. Specific shared features include elongated uropods decorated with actin filaments and spikes, trapped nuclear lobes, diminished leading edge size, small actin puncta throughout the cytosol that mediate actin flow, elevated RLC S19 phosphorylation, and diminished migration speed and directionality (9, 10, 14, 15, 47, 64, 65). Of note, maximum cell elongation and near total ablation of uropod retraction was achieved when infection was combined with Blebbistatin (Supplementary Video 10), which many indicate that *H. pylori* and Blebbistatin inhibit myosin IIA to different extents or at different stages of its activity cycle. Despite these many similarities, two differences were also identified. Blebbistatin-treated cells continued to ruffle and did not develop the anterior actin foci induced by *H. pylori*, and the subcellular localizations of RhoA and myosin IIA were somewhat different. What accounts for this is unknown, but more complex models that integrate effects of *H. pylori* on multiple targets should be considered. Candidates for further study include myosin IIA phosphatase which localizes to the leading edge and helps confine phosphorylated, active myosin IIA to the cell rear (9, 66) and the Rac and Cdc42 effector p21-activated kinase 2 (PAK2) which regulates leading edge adhesion and promotes frontness by preventing anterior accumulation of active RhoA and myosin IIA (67).

With future studies in mind, we also note that the main limitations of this study are the fact that our microscopy images are snapshots of dynamic processes in individual cells, whereas the western blotting data provide an average view of all cells in the population. In particular, we believe that the dynamic nature of PMN migration contributed to the heterogeneity of myosin IIA S19 phosphorylation detected by western blotting though the trends are conserved for all donors and replicates (**Figure 8A** and **Supplementary Figure 3**).

Inasmuch as the results of this study identify similarities between the effects of *H. pylori* and Blebbistatin they also exclude ROCKII inhibition as a key player in our migration defect. This conclusion is based on differences in neutrophil morphology and actin dynamics shown in **Figure 7** and the opposing effects of Y-27632 and *H. pylori* on myosin IIA S19 phosphorylation. Although not directly examined in this study, inhibition of myosin light chain kinase (MLCK) is also unlikely as cytosolic calcium was normal and infected PMNs were not defective for adhesion, rearward orientation of microtubules or trafficking of CD11b to the uropod, all of which are linked to MLCK *via* its myosin IIA-independent role in β2 integrin activation (52, 55, 68). Furthermore, disruption of MLCK function impairs uropod retraction only when neutrophils are plated on highly adhesive surfaces coated with extracellular matrix proteins such as fibronectin and vitronectin (55), conditions that were not used for any of the migration assays in this study.

Although chemotaxis of uninfected neutrophils has been extensively studied the effects of phagocytosis on this process have not. Published data show that neutrophils migrating toward fMLF, C5a or LTB_4_ arrest immediately after ingestion of yeast zymosan and do not exhibit further forward or reverse migration (69, 70). Our experimental design differed as we assayed chemotaxis 4 and 8 hours after phagocytosis. Under these conditions, prior infection with live *H. pylori* but not live *F. tularensis* or formalin-killed *H. pylori* inhibited chemotaxis toward fMLF as indicated by EZ-TAXIScan™ imaging. These data may indicate that phagocytosis does not invariably curtail neutrophil migration. On the other hand, it is also possible that immediate arrest is conserved but long-term chemotaxis inhibition is not. Thus, further studies of the kinetics and underlying mechanisms are warranted.

In keeping with the ability of *H. pylori* to thrive in a neutrophil-rich environment, several bacterial and host factors act in concert for robust recruitment of neutrophils to the infected human gastric mucosa including the neutrophil activating protein, the cecropin-like peptide Hp(2–20), urease and IL-8 (18, 19, 71). Although many leukocyte populations reach the lamina propria, neutrophils are unique in their ability to traverse the gastric epithelium. In this locale they become heavily infected, ingesting bacteria in the mucus layer, attached to epithelial surfaces and in gastric pits (18–20). Phagocytosis triggers a robust respiratory burst that is coupled to extracellular oxidant production and the associated tissue damage enhances nutrient availability, thereby favoring bacterial persistence, and neutrophil lifespan is prolonged (18, 19, 21, 29). The results of this study identify chemotaxis inhibition as a new aspect of N1-like subtype differentiation and *H. pylori*-infection and are also significant as they provide a potential explanation for the fact that infected PMNs do not disseminate to the spleen or other distal organs. Altogether, the data provide new insight into the mechanisms that underlie a chronic, PMN-dominant inflammatory response that can progress from gastritis to peptic ulcer disease or gastric cancer and directly impacts 50% of humans worldwide.

During *H. pylori* infection polarity and integrity of the gastric epithelium is compromised and this has been linked in *in vitro* studies to urease signaling and urease-derived ammonia as triggers of MLCK- and ROCKI-mediated myosin IIA activation and actin contraction (71, 72). In addition, ectopic expression of CagA or its introduction into unpolarized gastric epithelial cell lines by type IV secretion causes cell scattering and elongation, commonly referred to as the hummingbird phenotype, which is enhanced when combined with low concentrations of Blebbistatin (73, 74). Accordingly, it has been suggested that CagA may inhibit myosin IIA *via* effects on PAR-1 (73). In marked contrast, our data excluded essential roles for CagA, urease and the other virulence factors tested in dysregulation of infected PMN chemotaxis. This discordance is perhaps not surprising as epithelial cells and neutrophils differ markedly in structure and function. Moreover, ROCKI and ROCKII have distinct non-redundant roles that are in keeping with fundamental differences in mechanisms of amoeboid and mesenchymal motility (75, 76).

In summary, neutrophil chemotaxis is a highly regulated and dynamic process that requires efficient attractant sensing as well as spatiotemporal activation of Rho family GTPases and actomyosin dynamics for rapid amoeboid migration (7, 9). It has long been known that *H. pylori* uses a multifaceted strategy to recruit neutrophils to the gastric mucosa (18, 19). The results of this study demonstrate that once infected the ability of human neutrophils to chemotax is severely compromised, whereas other assayed aspects of cell function were unchanged. Our combined strategy of live video imaging, confocal microscopy and immunoblotting revealed a defect in uropod retraction that is driven by dysregulation of myosin IIA activity and F-actin dynamics and is coupled to aberrant subcellular localization of RhoA and ROCKII. Although additional studies are needed, our data advance understanding neutrophil plasticity in general and N1-like cells in particular. In the context of *H. pylori* pathogenesis our findings also suggest that the processes driving PMN accumulation in the infected gastric mucosa are more complex than previously appreciated.

## Data availability statement

The raw data supporting the conclusions of this article will be made available by the authors, without reservation.

## Ethics statement

The studies involving human participants were reviewed and approved by the Institutional Review Boards of the University of Iowa and the University of Missouri. All blood donors provided written informed consent to participate in this study.

## Author contributions

L-AA conceived of the study, designed experiments, analyzed data and co-wrote the manuscript. AP designed and performed experiments, analyzed data and co-wrote the manuscript. LK designed and performed experiments and analyzed data. LW provided EZ-TAXIScan™ expertise, designed and performed experiments and analyzed data. DI provided expertise, methods and chambers for the microfluidics experiments. All authors contributed to the article and approved the submitted version.

## Funding

This work was supported, in part, by funds from the US government including National Institutes of Health, NIAID R01 AI119965 awarded to L-AA, a postdoctoral fellowship *via* NIAID T32 AI007260 awarded to LW and a VA Merit Review Grant I01BX002108 awarded to L-AA. Part of this study was also supported by start-up funds awarded to L-AA by the University of Missouri School of Medicine.

## Acknowledgments

The authors acknowledge use of the University of Iowa Central Microscopy Facility and the assistance of Tom Moninger for imaging of microfluidic chambers. We also thank Dr. William Nauseef, University of Iowa, for sharing *S. aureus*.

## Conflict of interest

The authors declare that the research was conducted in the absence of any commercial or financial relationships that could be construed as a potential conflict of interest.

## Publisher’s note

All claims expressed in this article are solely those of the authors and do not necessarily represent those of their affiliated organizations, or those of the publisher, the editors and the reviewers. Any product that may be evaluated in this article, or claim that may be made by its manufacturer, is not guaranteed or endorsed by the publisher.
