# Supplementary material for: Helicobacter pylori-infected human neutrophils exhibit impaired chemotaxis and a uropod retraction defect
Source: Front Immunol. 2022 Oct 20;13:1038349. doi: 10.3389/fimmu.2022.1038349 (PMC9630475; doi:10.3389/fimmu.2022.1038349)
Supplement: Supplementary file 1 [file DataSheet_1.pdf]

Supplementary Video Legends

Video 1. EZ-TAXIScan^{TM} movie – PMNs migrating toward fMLF at 4 hours.

Video 2. EZ-TAXIScan^{TM} movie -- *H. pylori*-infected neutrophils migrating toward fMLF at 4 hours.

Video 3. EZ-TAXIScan^{TM} movie – PMNs migrating toward fMLF at 8 hours.

Video 4. EZ-TAXIScan^{TM} – movie -- *H. pylori*-infected neutrophils migrating toward fMLF at 8 hours.

Video 5. EZ-TAXIScan^{TM} movie -- Blebbistatin-treated PMNs migrating toward fMLF at 4 hours.

Video 6. EZ-TAXIScan^{TM} movie -- Blebbistatin-treated *H. pylori*-infected PMNs migrating toward fMLF at 4 hours.

Video 7. EZ-TAXIScan^{TM} movie – Y-27632-treated PMNs migrating toward fMLF at 4 hours.

Video 8. EZ-TAXIScan^{TM} movie – Y-27632-treated *H. pylori*-infected PMNs migrating toward fMLF at 4 hours.

Video 9. EZ-TAXIScan^{TM} movie -- Blebbistatin-treated PMNs migrating toward fMLF at 8 hours.

Video 10. EZ-TAXIScan^{TM} movie - Blebbistatin-treated *H. pylori*-infected PMNs migrating toward fMLF at 8 hours.

Video 11. EZ-TAXIScan^{TM} movie – Y-27632-treated PMNs migrating toward fMLF at 8 hours.

Video 12. EZ-TAXIScan^{TM} movie – Y-27632-treated *H. pylori*-infected PMNs migrating toward fMLF at 8 hours.

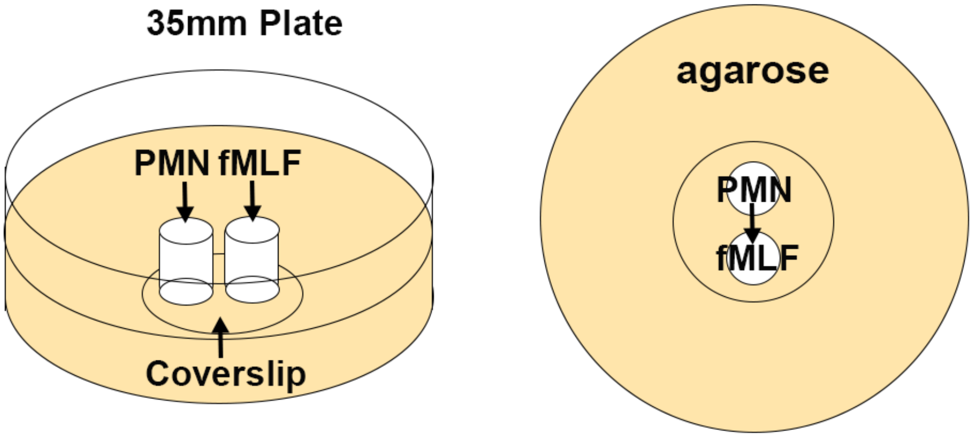

Supplementary Figure 1. Under agarose chemotaxis assay schematic. A MatTek dish with a central coverslip was overlaid with agarose. Two wells are cut in the agarose over the coverslip. Wells were then loaded with neutrophils or fMLF as indicated. PMNs migrate along the coverslip and under the agarose toward the fMLF containing well when incubated at 37°C.

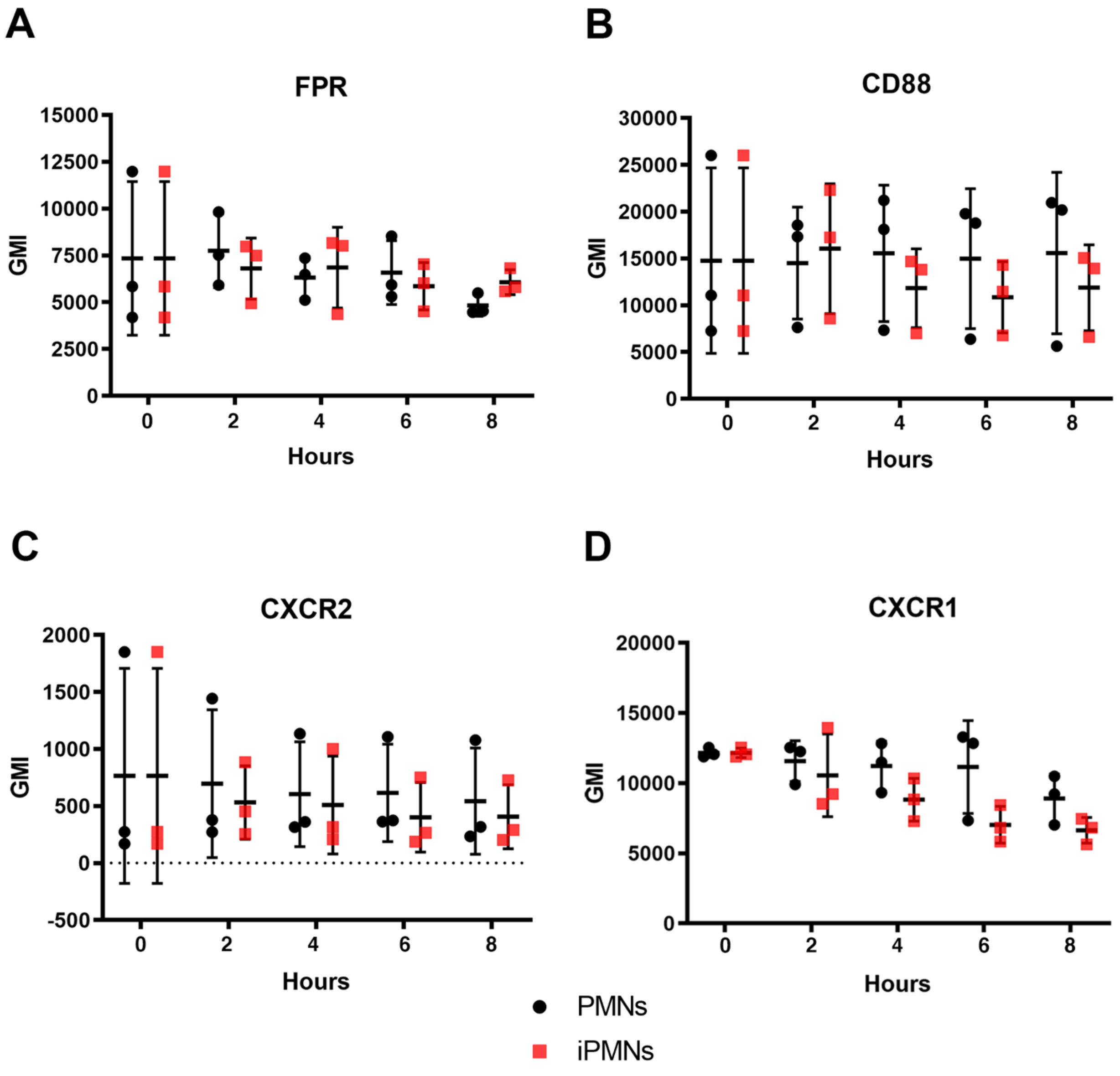

Supplementary Figure 2. Chemotactic receptor abundance is not significantly altered by *H. pylori* infection. Neutrophils were incubated at 37°C in the presence and absence of *H. pylori* for 0-8 hours, as indicated. At each time point surface chemotactic receptor abundance was quantified by flow cytometry. **(A)** Formyl peptide receptor (FPR). **(B)** CD88. **(C)** CXCR2. **(D)** CXCR1. Data are geometric mean intensity (GMI) ± SD, (n=3) and were analyzed by two-way ANOVA and Tukey's multiple comparison test. Differences between uninfected and infected PMNs (iPMNs) were not significant, $p>0.05$.

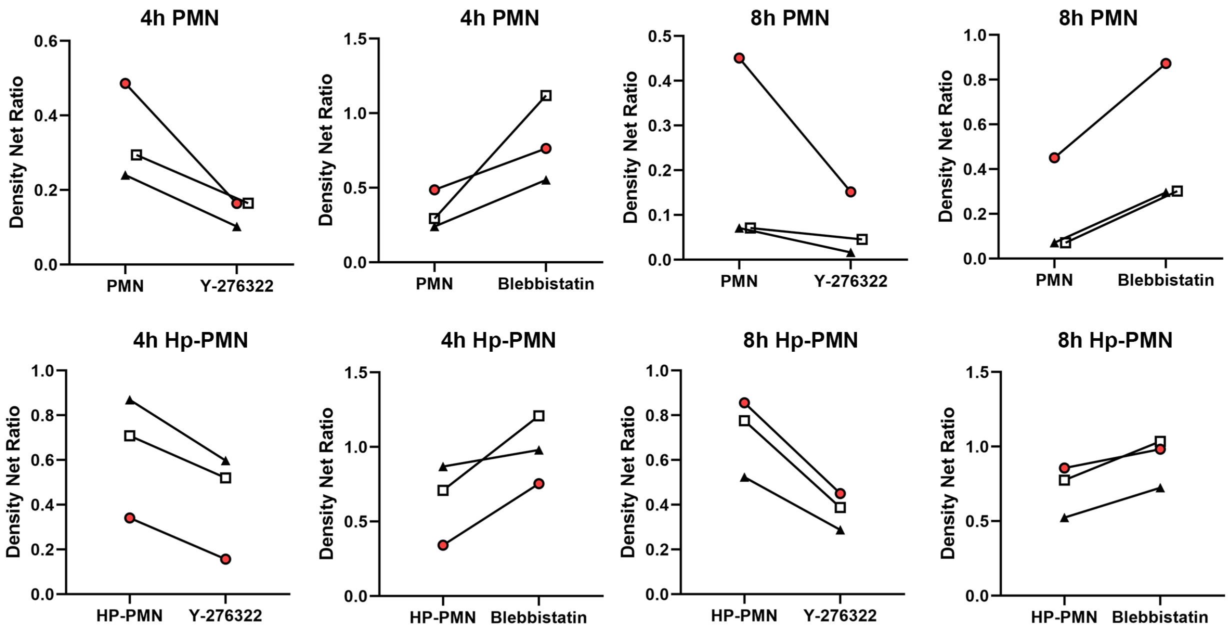

Supplementary Figure 3. Effects of infection, Y-27632 and Blebbistatin on myosin IIA RLC S19 phosphorylation. Phosphorylated S19 immunoblot band intensities were quantified and normalized to each GAPDH loading control using ImageJ. Graphs show data for untreated control or *H. pylori* (HP)-infected PMNs compared to their respective Y-27632 or Blebbistatin-treated counterparts at 4 and 8 hours. Symbols indicate results for individual blots shown in Figure 8A.

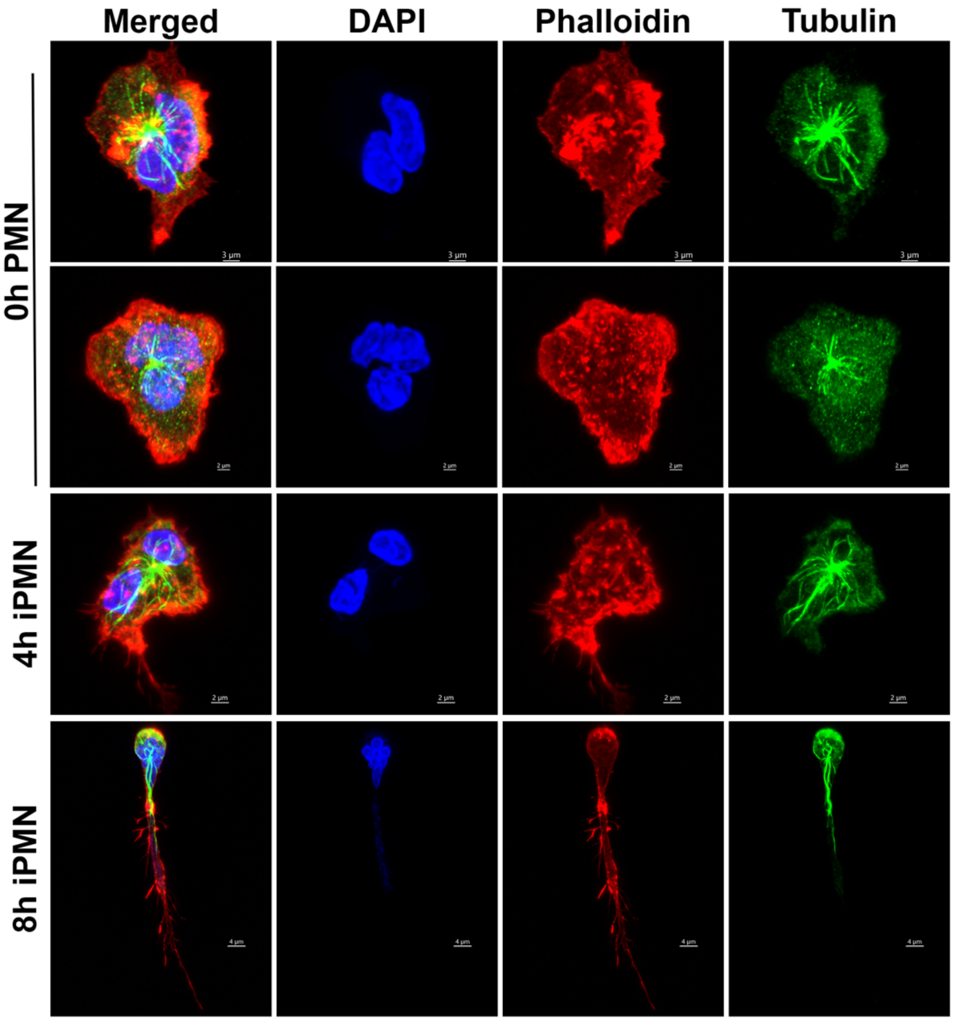

Supplementary Figure 4. Microtubules orient toward the uropod in control and infected neutrophils migrating toward fMLF. Confocal Z-stack images show the orientation and abundance of microtubules in cells migrating toward fMLF in MatTek dishes. Fixed cells were co-stained with DAPI to detect DNA (blue), rhodamine-phalloidin to detect F-actin (red) and antibodies to α-tubulin to detect microtubules (green). Images show the range of microtubule abundance and their subcellular localization relative to overall cell morphology in freshly isolated, uninfected cells (0h PMN) and cells at 4 and 8 hours after *H. pylori* infection (iPMN).

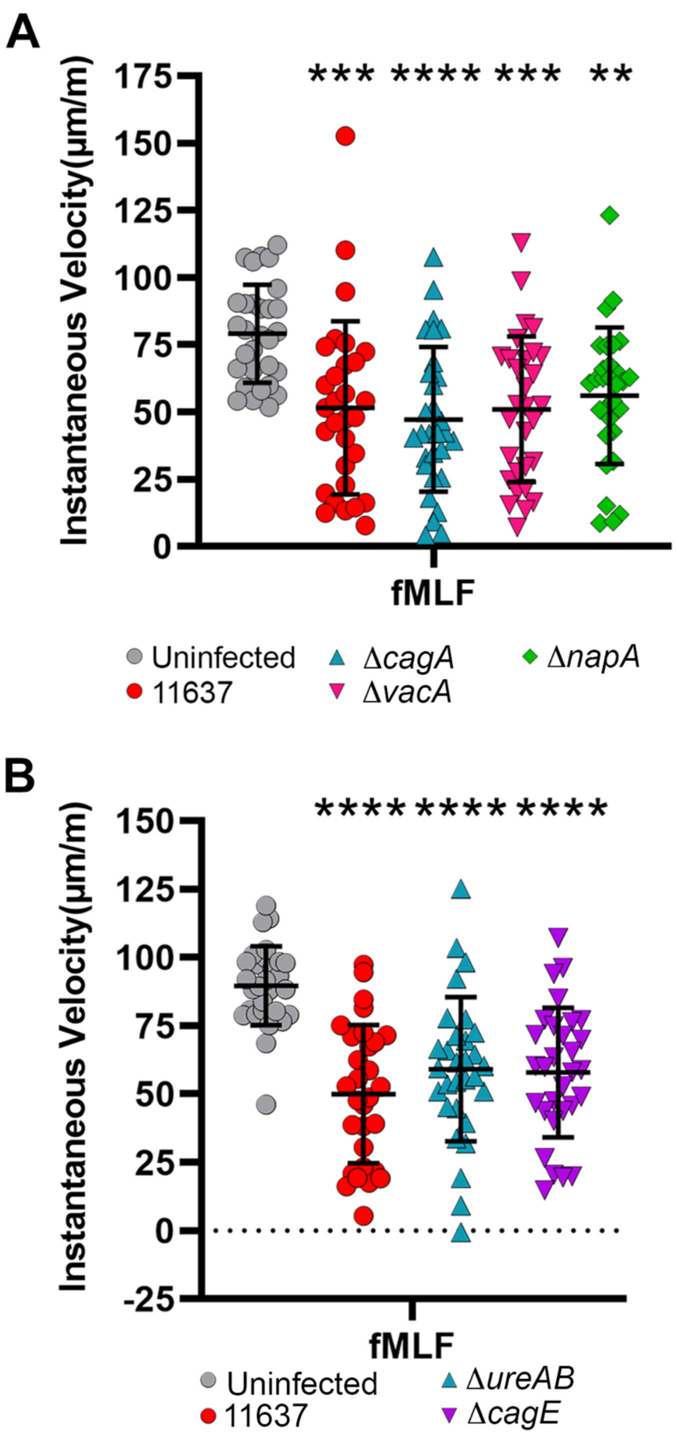

Supplementary Figure 5. All *H. pylori* mutant strains tested demonstrate impaired chemotaxis by EZTAXIScan. Neutrophils were left untreated or were infected with wild-type *H. pylori* 11637 or the isogenic mutant strains Δ*cagA*, Δ*vacA*, Δ*napA*, Δ*ureAB* or Δ*cagE*, as indicated. Chemotaxis toward fMLF was assayed by EZTAXIScan at 8 hours. Instantaneous velocity data are shown for individual cells (symbols) and as the mean $\pm$ SD of three determinations. ***$p<0.001$, ****$p<0.0001$ vs. uninfected control. Differences between mutant strains and wild-type *H. pylori* 11637 were not significant ($p>0.05$).

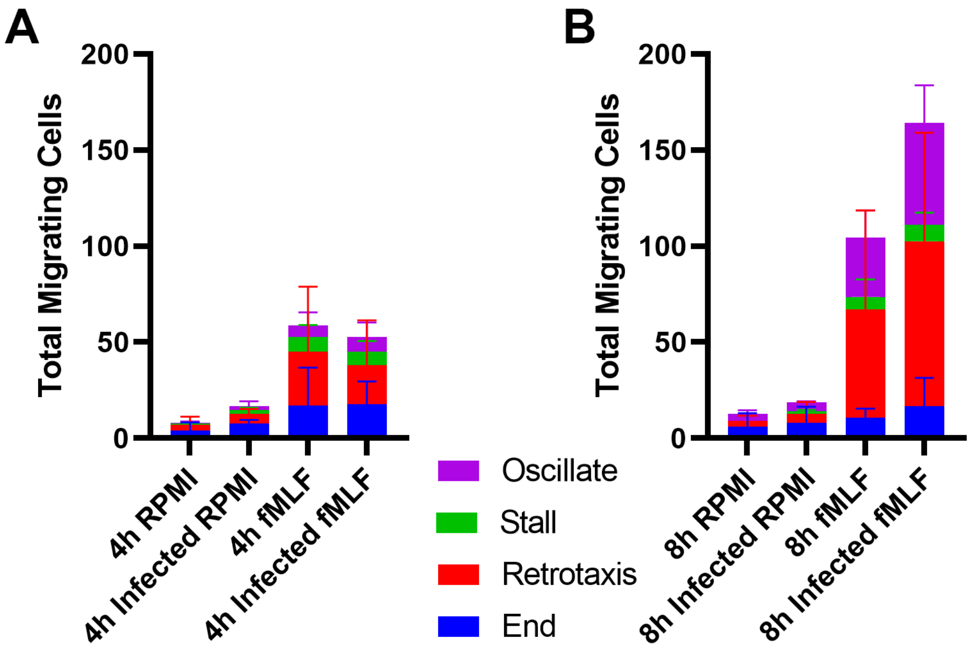

Supplementary Figure 6. Constricted 3D channel migration of *H. pylori*-infected PMNs toward fMLF is not impaired. Neutrophil migration toward fMLF was assayed in tapered channel microfluidic devices after infection with *H. pylori* or incubation at 37°C in medium alone (RPMI). Four cell migration behaviors were identified by live-cell imaging: forward and backward oscillation in the channel, stalled forward migration, reverse migration/retrotaxis or forward migration all the way to the end well containing fMLF. Devices were loaded for live cell imaging at 4 hours **(A)** or 8 hours **(B)** and behavior of individual cells was scored. Data are the mean + SD of two independent experiments.

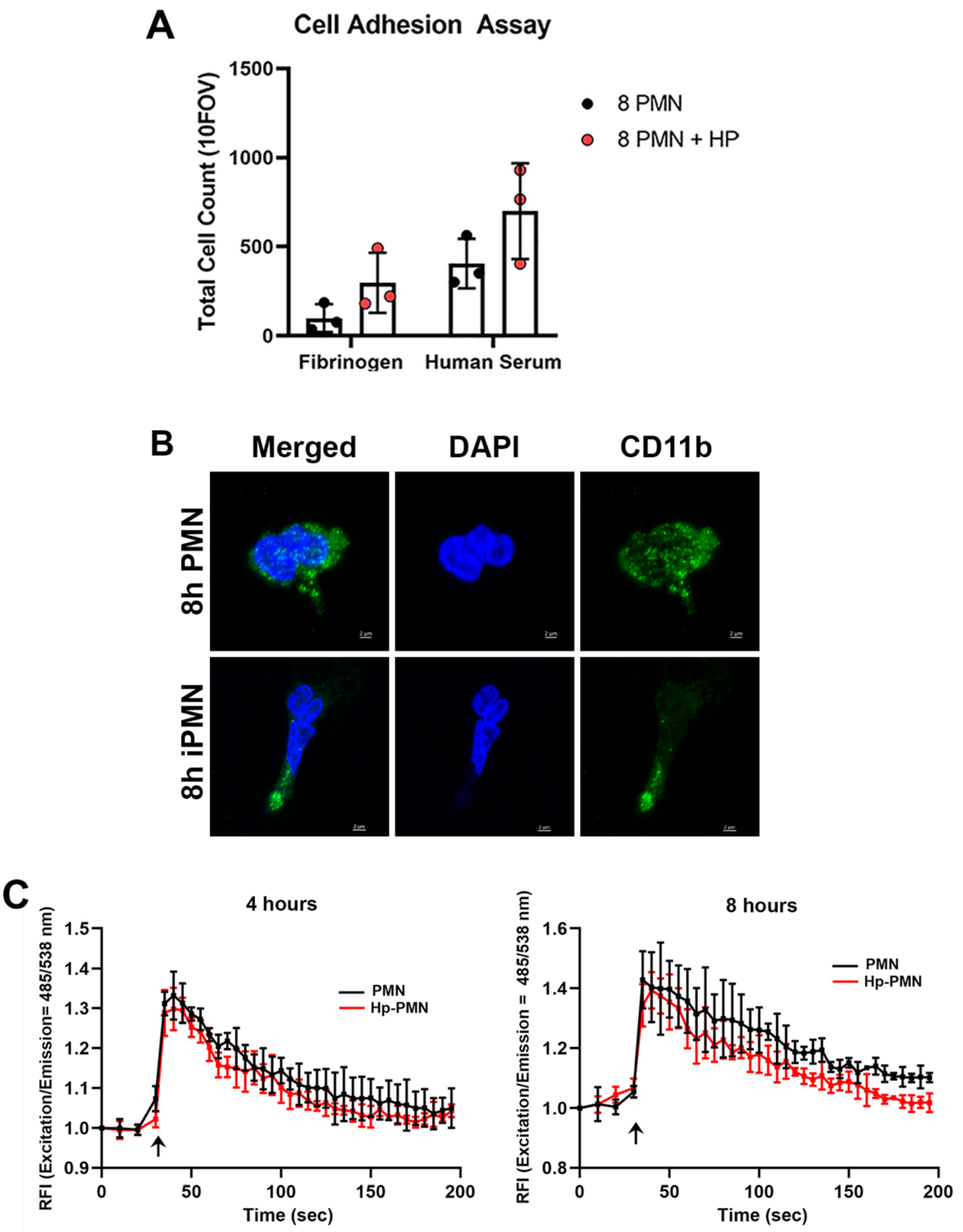

Supplementary Figure 7. Quantitation of neutrophil adhesion, CD11b localization and cytosolic Ca^{++}. (A) Control and *H. pylori*-infected neutrophils were plated on autologous human serum-coated or fibrinogen-coated glass coverslips and stimulated with fMLF for 5 min. Cells attached to each coverslip in 10 random fields of view (10FOV) were quantified by light microscopy after Hema-3 staining. Data are the mean ± standard deviation (n=3). **(B)** Representative confocal images show localization of CD11b in control and infected PMNs (iPMNs) at 8 hours. **(C)** Relative basal and fMLF-stimulated cytosolic calcium levels at 4 and 8 hours. Relative fluorescence intensity (RFI) of Fluo3-AM is shown. Vertical black arrows indicate injection of fMLF at 30 sec. Data are the mean ± SD of three independent experiments run in duplicate. Data did not significantly differ based on analysis by two-way ANOVA and Sidak multiple comparisons test (p>0.71). sec, seconds.

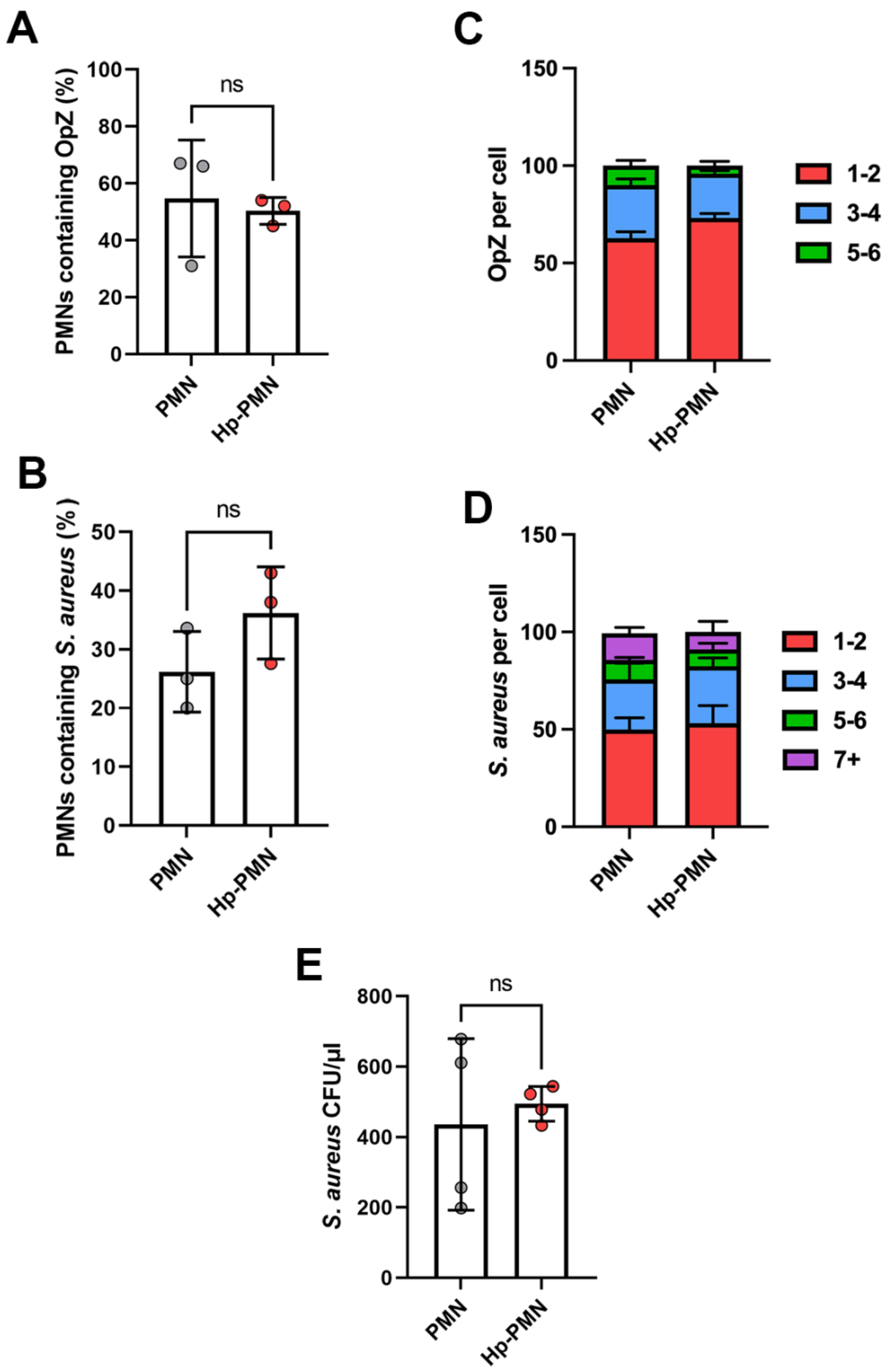

Supplementary Figure 8. *H. pylori* infection does not impair neutrophil phagocytic capacity or ability to kill *Staphylococcus aureus*. The ability of uninfected and *H. pylori*-infected PMNs to ingest serum-opsonized zymosan (OpZ) **(A-B)** or *S. aureus* **(C-D)** was quantified by light microscopy. **(A and C)** The percentage of cells that ingested at least one particle or bacterium. **(B and D)** Number of OpZ or *S. aureus* in each of 100 infected cells per experiment and condition. **(E)** Intracellular killing of *S. aureus* quantified by measurement of recovered colony forming units (CFU). In each case, data are the mean $\pm$ SD (n=3). Differences between control and infected PMNs were not significant (p>0.05).
